# Exercise rejuvenates microglia and reverses T cell accumulation in the aged female mouse brain

**DOI:** 10.1111/acel.14172

**Published:** 2024-05-15

**Authors:** Solal Chauquet, Emily F. Willis, Laura Grice, Samuel B. R. Harley, Joseph E. Powell, Naomi R. Wray, Quan Nguyen, Marc J. Ruitenberg, Sonia Shah, Jana Vukovic

**Affiliations:** ^1^ Institute for Molecular Bioscience, the University of Queensland Saint Lucia Queensland Australia; ^2^ School of Biomedical Sciences, Faculty of Medicine The University of Queensland Saint Lucia Queensland Australia; ^3^ Queensland Brain Institute, the University of Queensland Saint Lucia Queensland Australia; ^4^ Department of Psychiatry University of Oxford Oxford UK; ^5^ Oxford Big Data Institute, Li Ka Shing Centre for Health Information and Discovery University of Oxford Oxford UK

**Keywords:** active place avoidance, border‐associated macrophages, brain ageing, cognition, disease‐associated microglia, neuroinflammation, T cells

## Abstract

Slowing and/or reversing brain ageing may alleviate cognitive impairments. Previous studies have found that exercise may mitigate cognitive decline, but the mechanisms underlying this remain largely unclear. Here we provide unbiased analyses of single‐cell RNA sequencing data, showing the impacts of exercise and ageing on specific cell types in the mouse hippocampus. We demonstrate that exercise has a profound and selective effect on aged microglia, reverting their gene expression signature to that of young microglia. Pharmacologic depletion of microglia further demonstrated that these cells are required for the stimulatory effects of exercise on hippocampal neurogenesis but not cognition. Strikingly, allowing 18‐month‐old mice access to a running wheel did by and large also prevent and/or revert T cell presence in the ageing hippocampus. Taken together, our data highlight the profound impact of exercise in rejuvenating aged microglia, associated pro‐neurogenic effects and on peripheral immune cell presence in the ageing female mouse brain.

## Graphical abstract

Here we provide unbiased analyses of single‐cell RNA sequencing data, showing the impacts of exercise and ageing on specific cell types in the mouse hippocampus. We demonstrate that exercise has a profound and selective effect on aged microglia, reverting their gene expression signature to that of young microglia. Our data highlight the profound impact of exercise in rejuvenating aged microglia, associated pro‐neurogenic effects, and on peripheral immune cell presence in the ageing mouse brain.

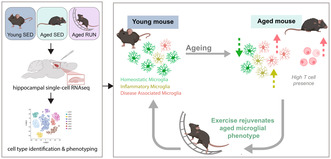

**Abbreviations**
ANOVA: analysis of variance
APA: active place avoidance
BAMs: border‐associated macrophages
DAMs: disease‐associated microglia
DCX: doublecortin
DEGs: differentially expressed genes
FACS: fluorescence‐activated cell sorting
FCS: foetal calf serum
FDR: false discovery rate
IP: intraperitoneal
LPS: lipopolysaccharide
LR: ligand receptor
NGS: normal goat serum
NPC: neural precursor cell
PBS: phosphate‐buffer saline
RUN: Runner
scRNAseq: single‐cell RNA sequencing
SD: standard deviation
SED: Sedentary
SYN: synaptophysin
UMAP: Uniform Manifold Approximation and Projection

## 1 INTRODUCTION

Ageing induces progressive physiological changes to multiple body systems over time. In humans, ageing is associated with a decline in both general physical wellbeing and cognitive abilities (Colcombe & Kramer, 2003; Josefsson et al., 2012; Persson et al., 2012). While it is recognised that elderly persons with mild cognitive impairments are more prone to develop dementia, there are few effective interventions for halting cognitive decline (Campbell et al., 2013; Mitchell & Shiri‐Feshki, 2008; Petersen et al., 2009; Yaffe et al., 2006). Observational studies in aged individuals consistently suggest that exercise can alleviate age‐related deficits in multiple physiological systems, including the brain (Colcombe et al., 2004; Heyn et al., 2004). Here, exercise can reverse grey matter loss in the hippocampus, an area important for learning and memory (Persson et al., 2012). While the mechanisms underlying these benefits remain poorly defined, exercise in general is known to increase neurotrophin expression, enhance synaptic plasticity and modulate the expression of various immune factors in the aged brain (Kramer et al., 2006; Neeper et al., 1995). Exercise also modulates the heightened ageing‐associated response of microglia to immune challenge (Barrientos et al., 2011; Hickman et al., 2013; Littlefield et al., 2015). Reported changes in microglial phenotypes following exercise include an increased expression of neurogenic factors such as brain‐derived neurotrophic factor (BDNF) and CX3CL1 and reduced expression of immune factors MHCII, IL‐1β and TNFα (Barrientos et al., 2011; Kohman et al., 2013; Littlefield et al., 2015; Vukovic et al., 2012).

In rodent studies, we and others have demonstrated that voluntary exercise can also mitigate age‐related cognitive impairment (Blackmore et al., 2021; Horowitz et al., 2020; Pietrelli et al., 2012; Speisman et al., 2013; van Praag et al., 1999; Wu et al., 2015). In mice, improvements in cognition are positively associated with exercise supporting the production of adult‐born neurons from neural precursors in the hippocampus (Marlatt et al., 2012; van Praag et al., 1999, 2005). Accordingly, neurogenesis has been proposed as a potential mechanism mediating exercise‐induced alleviation of age‐related cognitive impairment (Deng et al., 2010; Ramirez‐Amaya et al., 2006; Vukovic et al., 2013). However, the wider impacts of exercise on other cell types in the aged brain remain unclear.

Here, we have begun to address this gap in our understanding as to how exercise affects the brain, conducting single‐cell RNA sequencing (scRNA‐seq) and unbiased gene expression analyses to characterise the effects of both ageing and voluntary wheel running on the various cell types in the mouse hippocampus.

## 2 RESULTS

### 2.1 Single‐cell RNA sequencing identifies the effects of ageing and exercise on the mouse hippocampus

**FIGURE 1:**
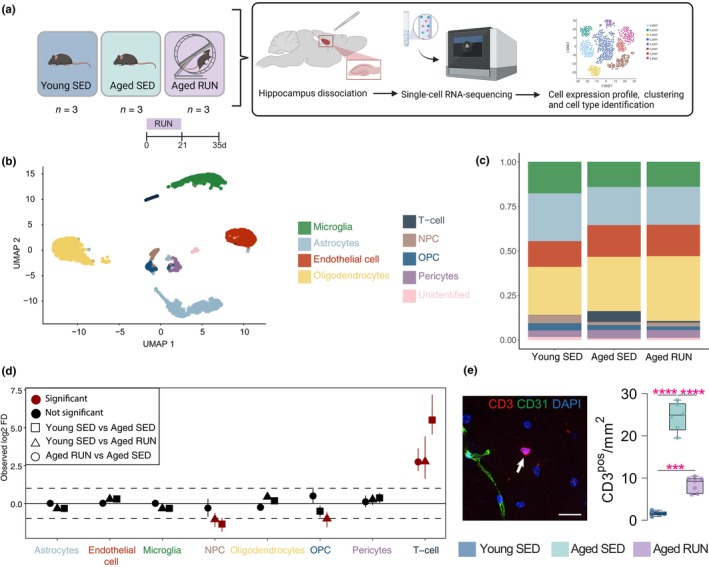
Single cell RNA‐sequencing of cell populations isolated from the young or aged brain following exercise. (a) Overview of experimental design. Cells were isolated from the hippocampi of young sedentary mice (3 months old, “Young SED”), aged sedentary mice (18 months old; “Aged SED”) or aged mice given 21 days of voluntary access to a running wheel (“Aged RUN”). In Aged RUN mice, the exercise period was followed by 14 days of rest prior to sacrifice. Single, live cells from dissociated hippocampi were sorted by FACS and processed to create single‐cell (sc)RNA‐seq libraries (*n* = 3 female littermates per condition). (b) Uniform Manifold Approximation and Projection (UMAP) plot of 9336 cells from the three experimental groups (Young SED, Aged SED, Aged RUN) that passed quality control thresholds (Figure S1). Cells clustered into nine distinct cell types, which were annotated based on their expression of canonical markers (as indicated). (c) Proportions of identified nine cell types in Young SED, Aged SED and Aged RUN mice. (d) Forest plot showing Log_2_FC of cell numbers between experimental conditions. The mean and 95% confidence interval (error bar; obtained from permutation) is shown for each cell comparison. Significance was obtained using a Monte Carlo permutation test with an FDR <0.05 and an absolute log change >1 (corresponding to a doubling of abundance compared to the reference group and represented as dashed lines). Note the age‐related increase in T cell numbers. (e) Independent validation of scRNA‐seq data showing increased T cell abundance in the ageing hippocampus. Representative confocal image (*left*) and quantification (*right*) of parenchymal CD3^pos^ T cells in the mouse hippocampus. Note that the number of CD3^pos^ T cells is increased in Aged SED mice compared to Young SED mice, and that access to a running wheel (i.e Aged RUN) significantly attenuates this change. Data points represent individual mice (*n* = 5–7 per group). Data are represented as mean ± SEM. Statistics: one‐way ANOVA, followed by Bonferroni post‐hoc comparison with Geisser–Greenhouse correction. ***p < 0.001, ****p < 0.0001.

We created scRNA‐seq libraries to assess the effects of ageing and interventional exercise on specific cell types present in the adult hippocampus. In the experiments described herein, female ‘young’ adult mice were 3 months old, while ‘aged’ mice were 18 months old. ‘Sedentary’ mice were housed in standard cages, while ‘runner’ mice had continuous access to a running wheel for a 21‐day period, followed by a 14‐day rest period prior to sacrifice. This exercise protocol was recently found optimal for reversing cognitive deficits in aged mice; the 14‐day rest period also allows exercise‐induced proliferating neural precursor cells to differentiate into immature hippocampal neurons (Blackmore et al., 2021). Cells obtained from the hippocampi of young sedentary (“Young SED”), aged sedentary (“Aged SED”) and aged runner (“Aged RUN”) mice (Figure 1a) passed stringent quality thresholds (Figure S1, see Methods), resulting in single‐cell transcriptome sequencing of a total of 9336 cells and 19,947 genes from nine mice (*n* = 3 per condition).

A total of nine distinct clusters of cells were identified (Figure 1b and Table S1). Compared to the Young SED condition, we found that ageing introduced significant changes in the proportion of T cells and neural precursor cells (NPCs). While T cells were scarce in Young SED mice, both Aged SED and Aged RUN mice had higher proportions of these cells (Figure 1c). We then used a Monte Carlo permutation test (see Methods) to validate and specifically compare the proportions of cells present between pairs of experimental groups (Figure 1d). Notably, compared to Young SED mice, this demonstrated an ~45‐fold increase in the number of T cells in Aged SED mice (Fold change (FC) = 45.57, False discovery rate (FDR) = 2.25 × 10^−04^), but only 6‐ to 7‐fold increase in Aged RUN mice (FC = 6.77, FDR = 1.8 × 10^−04^), indicating that T cell accumulation in the ageing brain is modulated by exercise. T cells were also the only cell type to show a significant difference in cell‐type proportion between the two aged conditions, with a significantly higher proportion of T cells observed in Aged SED mice compared to Aged RUN mice (FC = 6.68, FDR = 4.5 × 10^−04^). Further subsetting of these T cells identified distinct *Cd4*‐ and *Cd8a‐*expressing populations with a tissue‐resident memory T cell phenotype (*Cd69*, *Itgae*) (Mueller & Mackay, 2016) (Figure S2). Using orthogonal methods, we independently validated that the number of T‐cells was indeed significantly increased in the brains of Aged SED mice, both within the parenchyma and perivascular spaces, and that exercise prevented and/or reverted T‐cell presence in the ageing hippocampus (Figure 1e and Figure S3a,b); we similarly used immunostaining to confirm the presence of CD4^pos^ and CD8^pos^ populations in our aged mice, along with the mitigating effect of exercise thereon (Figure S3c,d). We lastly found that exercise also changed T‐cell abundance in at least one peripheral organ, namely the liver. Here, voluntary wheel running significantly reduced the number of CD3^pos^ T‐cells in aged RUN mice compared with aged SED mice (Figure S3e,f).

### 2.2 Exercise rejuvenates microglia in the ageing mouse hippocampus

**FIGURE 2:**
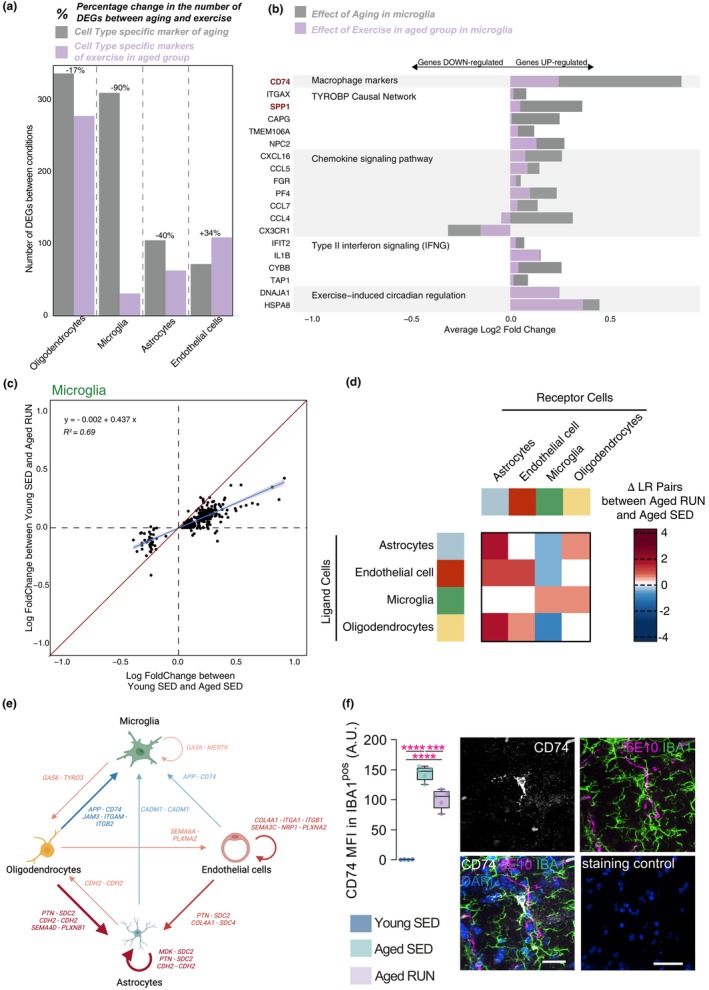
Exercise reverses the effect of ageing in microglia. (a) Bar plot showing the number of differentially expressed genes (DEGs) identified between Young SED and Aged SED mice (grey; gene markers of ageing), and between Young SED and Aged RUN (purple) in oligodendrocytes, microglia, astrocytes and endothelial cells. (b) Bar Plot showing the Log2FC in gene expression and their related pathways in Aged SED microglia (light grey; gene markers of ageing) and Aged RUN microglia (purple) compared to Young SED microglia. (c) Log fold change comparisons of gene expression in microglia. Each dot represents the log fold change of one of the DEGs identified between Young SED and Aged SED mice. The red line represents the equation x = y. The blue line corresponds to the best fit of the linear regression following the equation: log fold change (Young SED/Aged RUN) ~ log fold change (Young SED/Aged SED). Shaded area shows the 95% confidence interval of the fitted values. Coefficients, standard error and R^2^ for the linear regression are the following: 0.437*x* ± 0.012, R^2^ = 0.69. (d) Heatmap and diagram showing the number of significant ligand‐receptor (LR) pairs found between astrocytes, endothelial cells, microglia and oligodendrocytes from Aged SED and Aged RUN mice. (e) Schematic diagram showing specific LR pairs regulated with exercise in aged hippocampus, as predicted from cell–cell interaction analysis. Blue arrows show unique LR interactions that were significantly enriched in Aged SED but not Aged RUN mice; red arrows indicate significant LR pairs that were regulated by exercise in Aged RUN mice and not present in their Aged SED counterparts. (f) Validation of predicted changes in identified ligand‐receptor pair, APP‐CD74, in response to ageing and exercise. Note that CD74 expression in IBA1^pos^ cells is increased with ageing, but attenuated by exercise. CD74^pos^ cells were often found closely associated with APP^pos^ (i.e. 6E10‐stained) vascular structures and/or cells. Staining control (primary antibody omission) shows the absence of non‐specific secondary antibody binding. Scale bar = 50 μm. Statistics: one‐way ANOVA, followed by Bonferroni post‐hoc comparison with Geisser–Greenhouse correction. ***p < 0.001, ****p < 0.0001.

Next, we performed differential gene expression analysis to identify markers of ageing in each cell type (i.e. differentially expressed genes (DEGs) between Young SED and Aged SED mice). We then investigated how expression of these marker genes changed when exercise was introduced (i.e., between Aged SED and Aged RUN mice). The effect of exercise on markers of ageing was most pronounced in microglia compared to the three other main cell types in our dataset (i.e. astrocytes, endothelial cells, oligodendrocytes) (Figure 2a). A total of 310 markers of ageing were identified within microglia (Table S2). Of those genes, 90% were no longer differentially expressed in Aged RUN versus Young SED mice, where only 31 DEGs were identified in total (28 of these were previously identified as markers of aging; Table S3). We then conducted a closer examination of the effect of exercise on the 310 DEGs that we identified as markers of ageing. The majority of these genes (271 out of 310 genes; 90%) were upregulated with ageing (Figure S4a,b), and exercise reversed the age‐related increase in gene expression (Figure S4c). Finally, we also performed a downsampled analysis to confirm these results, ruling out any potential confounding effects relating to differences in cell number between conditions and/or clusters. We observed an inevitable decrease in the number of significant DEGs due to information loss (Figure S5a), but a near‐perfect correlation between the calculated Log fold change for each gene before and after downsampling (Figure S5b). We can therefore conclude that the observed gene expression changes with ageing and exercise are not influenced by any differences in cell number between conditions (or clusters). Taken together, exercise ameliorated age‐related changes in gene expression, such that the transcriptional profile of microglia from aged exercising mice resembled that of young mice.

Pathway analysis for the identified markers of ageing in microglia showed that many of these were linked to immune processes (Figure 2b; TYROBP causal network: FDR corrected *p* = 2.63 × 10^−02^; Type II interferon signalling: FDR corrected *p* = 8.74 × 10^−03^ and Chemokine signalling pathway: FDR corrected *p* = 2.63 × 10^−02^). This contrasts with only two significant pathways identified from the Young SED versus Aged RUN comparison (Macrophage markers: FDR corrected *p* = 9.78 × 10^−04^ and Exercise‐induced circadian regulation: FDR corrected *p* = 2.18 × 10^−02^). We investigated the changes in genes underlying those pathways. Out of the 19 microglial genes identified within significant pathways, most were upregulated with ageing (Aged SED vs. Young SED); this effect was less pronounced with running (Aged RUN vs. Young SED). Using orthogonal methods, we already validated changes in the expression of microglial Spp1 (gene most upregulated with ageing; Figure S4d) and CD74 (gene with the greatest change in response to ageing; Figure 2f). Taken together, we conclude that exercise counters the effect of ageing on the transcriptional profile of microglia.

We next performed linear regression to further compare the expression fold changes for ageing‐related genes in the Young SED versus Aged SED (*x*‐axis) with those observed in the Young SED versus Aged RUN comparison (*y*‐axis). Here, we would expect a regression coefficient closer to 0 if exercise reverses these age‐related gene expression changes. Conversely, if exercise were to have minimal effect on these putative ageing biomarkers, then we would expect to see a regression coefficient and R^2^ (a measure of goodness of fit) close to 1 (that is, gene expression differences in aged vs. young mice being similar with or without exercise). Microglia displayed a strong effect of exercise with a regression coefficient closer to 0 than 1 (0.44 ± 0.01, R^2^ = 0.69; Figure 2c); downsampled analyses yielded the same outcome (Figure S5c–f) and a very similar regression coefficient for microglia (0.39 ± 0.01). Similar analyses performed on other cell types (astrocytes, oligodendrocytes and endothelial cells) highlighted that this effect of exercise on microglia is unique (see Supplementary note 1 in Data S1). Thus, unlike other cell types in our dataset, exercise seems to rejuvenate aged microglia, reverting their transcriptional profile towards the young state.

To further validate the effect of exercise on the ageing profile in microglia, we took advantage of the previously published dataset by Hammond et al. (2019), which explored the microglia transcriptome throughout the mouse lifespan. scRNAseq libraries from Hammond et al. (2019) include those from young adult (postnatal day 100) and aged (postnatal day 540) male mice, which closely matched the ages of animals used in this study. A total of 1362 markers of ageing were identified between these two ages (Figure S6 and Table S4), 247 (80%) of which overlapped with genes that also showed significant enrichment as DEGs in our dataset.

We next investigated how the externally identified markers of ageing from Hammond et al. (2019) behaved in our own dataset, comparing the reported fold‐changes for these 1362 genes to those observed in our own Young SED versus Aged SED comparison. Fitting a linear regression to these data returned a regression coefficient of 0.30 ± 0.03, R^2^ = 0.45, with many of the identified markers of ageing behaving similarly in both datasets (Figure S6f). In contrast, a similar linear regression analysis against Young SED versus Aged RUN comparison returned a coefficient of 0.12 ± 0.02, R^2^ = 0.23 (Figure S6g), indicating that exercise also influenced the expression of these externally identified gene markers of ageing. Taken together, these findings independently validate both the observed effect of ageing on the microglial transcriptome and the specific attenuating effect of exercise (as shown in Figure 2c) thereon.

### 2.3 Cell–cell interaction analysis highlights reduced signalling to microglia with exercise

Having established the effects of ageing and interventional exercise on the transcriptome of individual cell types, we next investigated interactions that were uniquely enriched between them. We focused our analysis here on significant ligand receptor (ΔLR) pairs identified between the Aged RUN and Aged SED conditions (Figure 2d–f; Table S5). Microglia were the only cell type with a predicted reduction in input received when exercise is present, with fewer signals received from oligodendrocytes (ΔLR = −2; *APP/CD74*, *JAM3/ITGAM/ITGB2*), astrocytes (ΔLR = −1; *CADM1/CADM1*) and endothelial cells (ΔLR = −1; *APP/CD74*). We already used immunofluorescence to validate changes in *APP/CD74* with ageing and exercise, highlighting the close relationship between CD74 on microglia and APP staining (Figure 2f).

In contrast to microglia, astrocytes were predicted to receive increased input from both endothelial cells (ΔLR = 2; *PTN/SDC2*, *COL4A1/SDC4*) and oligodendrocytes (ΔLR = 3; *PTN/SDC2*, *CDH2/CDH2*, *SEMA4D/PLXNB1*) when exercise was introduced. Endothelial cells followed the same pattern, with cell–cell interaction analysis predicting increased signalling from oligodendrocytes (ΔLR = 1; *SEMA6A/PLXNA2*) following exercise. Oligodendrocytes also received increased input from astrocytes (ΔLR = 1; *CDH2/CDH2*) and microglia (ΔLR = 1; *GAS6/TYRO3*). Amidst these various unique cell–cell interactions, microglia again stand out as having a ‘stand‐alone’ reaction, in that they received a reduced rather than increased input in response to exercise.

### 2.4 Microglial subtypes and border‐associated macrophages (BAMs) are differentially affected by ageing and exercise

**FIGURE 3:**
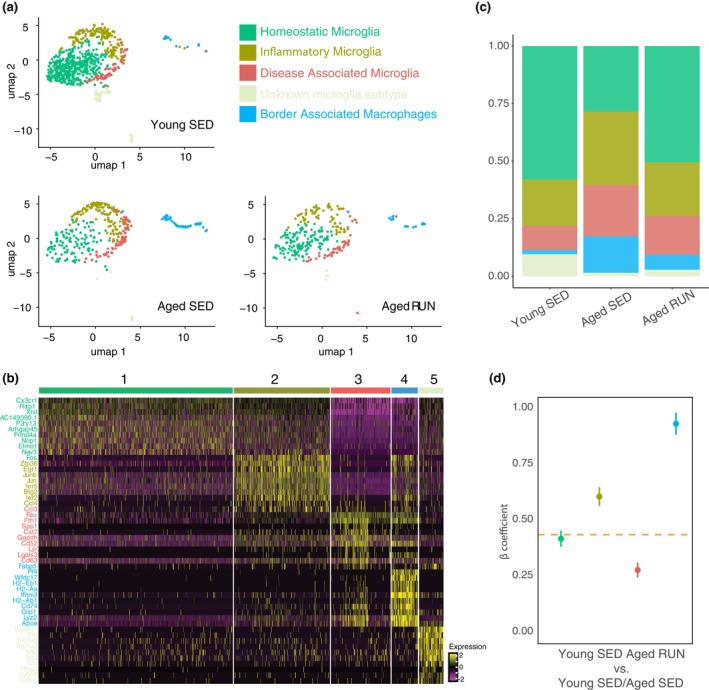
Microglial subtypes are differentially impacted by ageing and exercise. (a) UMAP plots showing the five identified sub‐clusters of brain myeloid cells, namely homeostatic microglia (green), inflammatory microglia (brown), disease‐associated microglia (DAM; red), an unknown microglia subtype (light grey), and border‐associated macrophages (blue) for the different experimental groups (Young SED, Aged SED and Aged RUN). (b) Heatmap of the top gene markers for each sub‐cluster. A full list of genes and publications of origin can be found within Table S6. (c) Stacked bar chart showing the proportion of each microglial/myeloid cell subtype for each of the experimental groups. (d) Dot plot representing coefficients of linear regressions fitted to each cluster using the 310 DEGs identified between the Young SED and Aged SED condition. The orange dashed line represents the coefficient identified when all subtypes were combined (Coefficients and standard error: 0.437 ± 0.012). Dot colours reflect microglia subtypes and/or states and BAMs, as specified in Figure 3a. Coefficients and standard errors are: 0.419 ± 0.036 (homeostatic microglia; green), 0.607 ± 0.042 (inflammatory microglia; brown), 0.278 ± 0.033 (disease‐associated microglia; red) and 0.932 ± 0.049 (BAMs, blue).

Having identified microglia as the cell type that was most responsive to exercise, we next assessed how ageing and/or exercise may affect their activation state(s). Here, we performed a principal component analysis followed by UMAP clustering to identify phenotypically‐distinct sub‐clusters of myeloid cells in the mouse hippocampus (Figure 3a), defined by their expression of specific markers (Figure 3b, Table S6). We identified five distinct cellular sub‐clusters, four of which were bona fide sub‐clusters of microglia representing different states (i.e. sub‐cluster 1: homeostatic; sub‐cluster 2: inflammatory; sub‐cluster 3; disease‐associated; and sub‐cluster 4. unknown/not previously described). Sub‐cluster 5 was found to represent border‐associated macrophages (BAMs), a small population of specialised macrophages that reside in the choroid plexus, meningeal and perivascular spaces (Goldmann et al., 2016; Mrdjen et al., 2018; Van Hove et al., 2019).

We first examined the effect of ageing on the different microglial subtypes and/or states. Here we found a lower proportion of homeostatic microglia (FC = 0.49, FDR = 9.99 × 10^−05^) in Aged SED compared to Young SED mice, while inflammatory microglia and disease‐associated microglia (aka DAMs) were relatively more abundant (inflammatory: FC = 1.59, FDR = 9.99 × 10^−05^; disease‐associated: FC = 2.13, FDR = 9.99 × 10^−05^) (Figure 3c). The proportion of cells in sub‐cluster 4, representing a not previously defined microglial phenotype, was also significantly lower in Aged SED compared to Young SED mice (FC = 0.15, FDR = 9.99 × 10^−05^). The age‐dependent shift in the proportions of microglial phenotypes towards more inflammatory and/or disease‐associated states was attenuated with exercise. Specifically, we found that the Aged RUN group had a higher proportion of homeostatic microglia compared to the Aged SED group (FC = 1.79, FDR = 2.50 × 10^−04^), and there were only small differences between the proportions of homeostatic microglia and disease‐associated microglia when comparing Young SED and aged RUN mice (homeostatic: FC = 1.15, FDR = 1.97 × 10^−02^; disease‐associated: FC = 0.62, FDR = 7.50 × 10^−03^). Overall, the relative presence of microglial states present in Aged RUN mice more closely resembled those of Young SED mice, providing further evidence that exercise can ameliorate the maladaptive microglial phenotypes that typically become more prevalent with ageing. This exercise‐induced ‘rejuvenation’ of microglial states was further corroborated by RNA velocity analysis (La Manno et al., 2018) (Figure S7), which indicated a transition in phenotype from inflammatory and disease‐associated microglia towards the more homeostatic transcriptional state in Aged RUN mice.

Next, we investigated if the restorative transcriptional changes after exercise were observed in all microglia sub‐clusters or more select states. For this, we examined how exercise affected identified gene markers of ageing in individual microglia sub‐clusters relative to the average gene expression levels calculated across all microglial cells (Figure 3d). In this analysis, a coefficient of 1 would again indicate no influence of exercise (i.e. DEG fold changes in Aged SED vs. Young SED are similar to fold changes in Aged RUN vs. Young SED), while a coefficient lower than that calculated for all microglia combined (0.44 ± 0.01) would indicate this particular microglial state to be a key driver behind any observed impact of exercise over the microglial transcriptome. While all microglial sub‐clusters responded to exercise, it was the disease‐associated microglia that had the lowest coefficient (0.28 ± 0.03); homeostatic (0.42 ± 0.04) microglia had a coeffiecient equivalent to the observed average. On the other hand, inflammatory microglia (0.61 ± 0.04) had higher coefficients, showing that they are less impacted by exercise than the average. Our data thus demonstrate a differential effect of exercise on different microglial states, highlighting also that disease‐associated microglia appear most responsive to exercise. Collectively, based on both the significant changes in the relative proportion of disease‐associated microglial cells (Figure 3c), and their dramatic transcriptional response to exercise, we identify disease‐associated microglia as the sub‐cluster and/or state that is making the greatest contribution towards the overall rejuvenation of the microglial transcriptome following exercise.

Beyond the analyses of bona fide microglial sub‐clusters, we also examined age‐related changes in BAMs, the fifth sub‐cluster of myeloid cells we identified by principal component analysis and subsequent UMAP clustering (Figure 3a). The proportion of BAMs was dramatically higher in Aged SED than Young SED mice (FC = 8.4, FDR = 9.99 × 10^−05^; Figure 3c), to the extent that these cells exhibited the biggest change in relative abundance of all the sub‐clusters examined. Notably, exercise had a significant attenuating effect on the over‐representation of BAMs in Aged SED mice (FC = 2.46, FDR = 2.50 × 10^−04^). We independently confirmed these results through orthogonal methods, finding that BAM numbers were indeed increased in Aged SED mice and that this change was attenuated by exercise (Figure S8a–d). That said, the expression of markers of ageing in these cells remained largely unaffected by exercise at the transcriptome level (Figure 3d), returning a coefficient of 0.93 ± 0.05 when the effect of exercise on ageing‐related DEGs in the BAM sub‐cluster was compared to the average gene expression levels of all sub‐clusters. This finding was in stark contrast to the microglial sub‐clusters, all of which underwent transcriptional changes in response to exercise. Taken together, these findings indicate that BAMs and microglia respond differently to both ageing and exercise.

### 2.5 Microglia are required to support exercise‐induced neurogenesis in the aged brain

Previous studies, including our own, established that exercise improves cognitive function in aged rodents (Horowitz et al., 2020; Speisman et al., 2013; Van Praag et al., 2005; Vukovic et al., 2012). Having identified here that aged microglia display the greatest transcriptional responsiveness to exercise, we next aimed to determine the extent to which these cells influence the cognitive abilities of aged mice under both basal and exercise conditions. We also examined whether microglia mediate exercise‐induced adult neurogenesis in aged mice (Blackmore et al., 2021; Vukovic et al., 2012).

**FIGURE 4:**
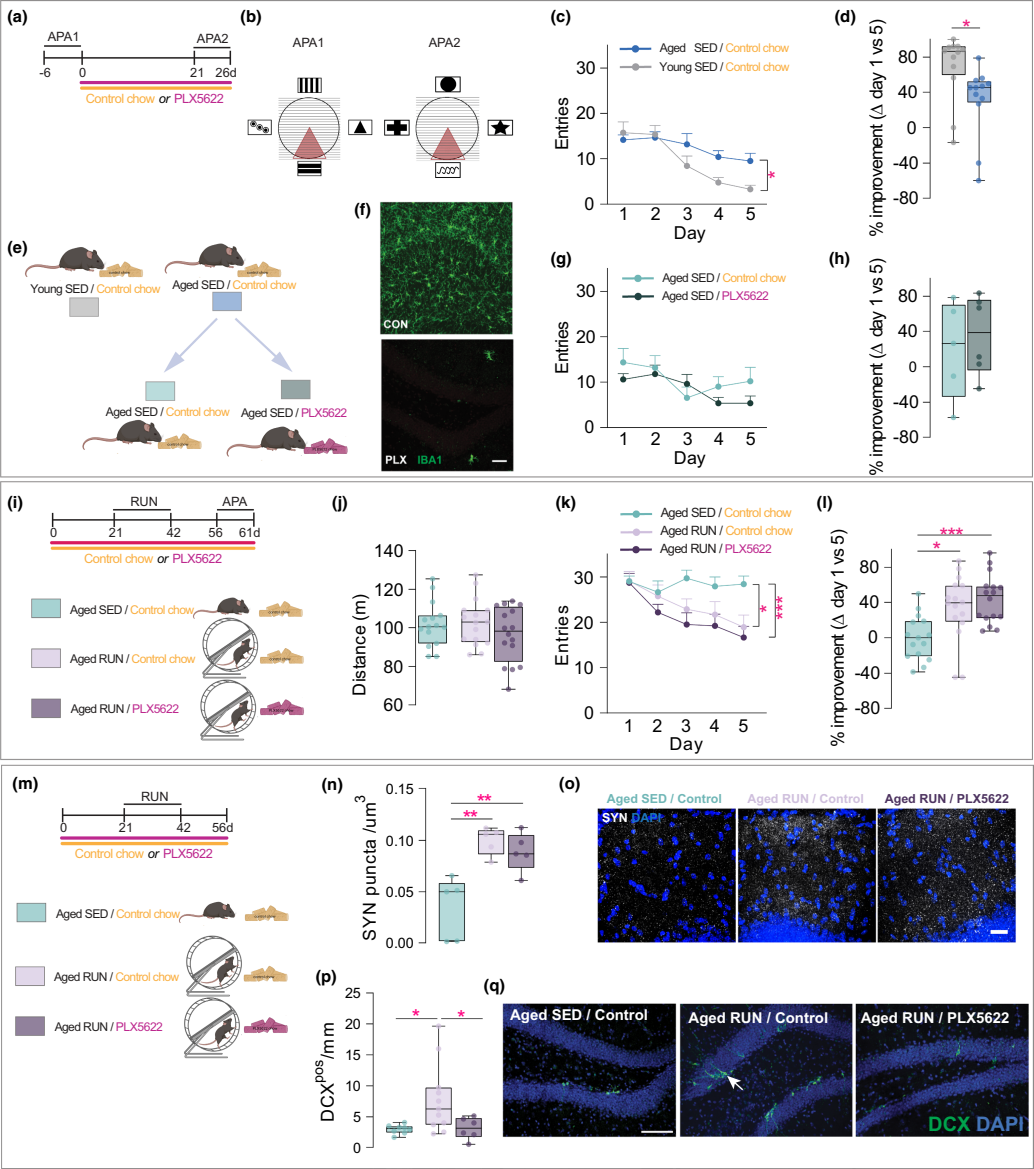
Benefits of exercise on cognitive abilities of aged mice remain with microglial depletion. (a) Overview of experimental timeline. Young adult (3‐month‐old) and aged (18‐month‐old) mice underwent active place avoidance (APA) testing over a course of 5 days (APA1). Aged mice were then placed on PLX5622‐containing chow (to deplete microglia), or control chow and re‐tested for acquisition of a new APA task (APA2) 21 days later. (b) Diagram of the visual cues used during APA testing prior to (APA1) and after (APA2) PLX5622 (or control chow) administration. The maroon triangle indicates the shock zone location. Note that different visual cues were used in APA2 versus APA1 to assess spatial learning (as opposed to task recall). (c) Shock zone entries during APA1 testing (10‐min trials/day). Aged mice had significantly more entries on day 5 compared to young adult mice (198% increase, *t*(115) = 2.79, *p* = 0.031, *n* = 12–13). (d) Percentage improvement in APA1 performance of individual mice (*t* = 2.50, df = 23, *p* = 0.012; minimum, 25% percentile, median, 75% percentile, maximum, Young SED: −16.67, 59.70, 86.34, 91.96, 100.00; Aged SED: −60.00, 24.16, 45.45, 51.67, 78.95, 138.90). (e) Schematic overview showing the split of aged sedentary mice used in APA1 into two groups, receiving either control chow (yellow), or PLX5622‐containing chow (to deplete microglia, pink). (f) Confocal images of IBA1 immunostaining showing effective depletion of microglia in the hippocampus of aged (18‐month‐old) mice that were fed PLX5622 (PLX) or control (CON) chow. Scale bar: 50 μm. (g) Entries into the shock zone during APA2 (10‐min trial/day) for aged mice given either control of PLX5622 chow (F[1,9] = 0.075, *p* = 0.79). (h) Percentage improvement in APA2 testing for aged mice with and without microglia (*t* = 0.31, df = 10, *p* = 0.77; minimum, 25% percentile, median, 75% percentile, maximum, Aged SED/control chow: −57.14, −33.83, 26.67, 70.38, 78.26; Aged SED/PLX5622: −169.20, −25.00, 11.11, 73.33, 83.33). (i) Overview of experimental layout and timeline. Aged (18‐month‐old) mice received either control or PLX5622‐containing chow (to deplete microglia) for 61 days. Mice were allowed to run for 21 days, allowed a 2‐week rest period, and then tested in APA 14 days after completion of the exercise paradigm. (j) Distance travelled during the habituation trial of APA testing (shock zone off; minimum, 25% percentile, median, 75% percentile, maximum, Aged SED/control chow: 85.21, 92.01, 100.5, 106.40, 125.30; Aged RUN/control chow: 86.10, 92.72, 103.00, 109.20, 127.40; Aged RUN/PLX5622 run: 68.33, 82.44, 98.38, 111.0, 114.0). (k) Total number of entries into the shock zone during APA testing (20‐min trial/day; F(2,44) = 3.81, *p* = 0.0297). (l) Percentage improvement in APA performance for individual mice, assessed by the change in entries on testing day 5 versus day 1 for individual mice (F[2,44] = 8.64, *p* = 0.0007; minimum, 25% percentile, median, 75% percentile, maximum, Aged SED/control chow: 38.46, −20.83, 0, 18.75, 50.0; Aged RUN/control chow: −44.44, 18.63, 39.57, 59.24, 86.96; Aged RUN/PLX5622: 7.69, 22.70, 47.70, 58.17, 96.30). (m) Schematic of experimental timeline, as detailed above, used to examine neuroplasticity and neurogenic effects of exercise. Aged (18‐month‐old) mice received either control or PLX5622‐containing chow (to deplete microglia) for 56 days. Mice were allowed 14 days of rest after 21 days of voluntary wheel running to allow newborn cells to differentiate into DCX^pos^ immature neurons. (n) Exercise (RUN) increased the number of synaptophysin (SYN) puncta in the hippocampus of 18‐month‐old mice compared to sedentary (SED) aged‐matched controls (F(2,12) = 12.88, *p* = 0.0010). PLX5622 did not significantly alter the increase in SYN observed in Aged RUN mice (Aged SED/Control chow vs Aged RUN/PLX5622, *p* > 0.99; *n* = 5/group; minimum, 25% percentile, median, 75% percentile, maximum: Aged SED/Control chow: 0.0016, 0.0018, 0.0496, 0.058, 0.065; Aged RUN/Control chow, 0.079, 0.086, 0.106, 0.110, 0.112; Aged RUN/PLX5622: 0.061, 0.073, 0.087, 0.105, 0.112). (o) Representative confocal images for synaptophysin (SYN) staining in the hippocampus of Aged SED/Control chow, Aged Run/Control chow and Aged RUN/PLX5622 mice. Scale bar = 50 μm. (p) Exercise (RUN) increased the number of immature DCX^pos^ neurons in 18‐month‐old mice treated with a control diet compared to sedentary age‐matched controls (SED; 2.64‐fold increase, *t*(26) = 2.84, *p* = 0.017, *n* = 8–11). PLX5622 significantly decreased the number of DCX^pos^ immature neurons in mice that underwent exercise (2.50‐fold decrease, *t*(26) = 2.81, *p* = 0.019, *n* = 6–11; minimum, 25% percentile, median, 75% percentile, maximum, Aged SED/control chow: 1.69 2.43, 3.07, 3.44, 4.05; Aged RUN/control chow, 2.25, 3.68, 6.23, 9.71, 19.69; Aged RUN/PLX5622: 0.57, 1.72, 3.16, 4.74, 5.13). (q) Representative confocal images of immature DCX^pos^ neurons in hippocampus of aged mice fed either control or PLX5622‐containing chow. Scale bar = 50 μm. Note the exercise‐induced increase in DCX^pos^ cells in the RUN condition, and the absence of this when microglia were depleted (PLX5622). Data are represented as mean ± SEM unless specified otherwise. Statistics: unpaired Student's *t*‐test (d, h), repeated two‐way ANOVA (c, g, k), or one‐way ANOVA (j, l, n, p) both followed by Bonferroni post‐hoc comparison with Geisser–Greenhouse correction. *p < 0.05, **p < 0.01, ***p < 0.001. Data points represent individual mice.

We first determined the baseline cognitive abilities of young (3‐month‐old) and aged (18‐month‐old) mice in the active place avoidance (APA), a challenging hippocampal‐dependent spatial learning and memory task in which animals must navigate a rotating turntable and change their location continuously based on visual cues to avoid encountering a stationary shock zone (Vukovic et al., 2013; Willis et al., 2017, 2020) (Figure 4a,b). All mice explored the testing arena to a similar extent during habituation (shock zone turned off), indicating that locomotor abilities were not different between groups (data not shown and Figure 4j); the number of entries into the shock zone (‘virtual shocks’) was also not different here between experimental groups (Young SED: 57 ± 2 vs. Aged SED: 53 ± 4; *p* = 0.53). During baseline APA testing (APA1), Young SED mice (on control chow) quickly learned to avoid the shock zone, as evident from a reduction in shock zone entries over five consecutive days of testing (i.e., improved performance; Figure 4c,d). In sharp contrast, Aged SED mice (on control chow) made significantly more mistakes (i.e., greater number of entries into the shock zone) than their younger counterparts; they also failed to show a clear improvement in performance over time, indicating that they were unable to learn the APA task (Figure 4c,d).

To test the role of microglia in spatial learning and memory abilities of aged mice, we next used the CSF‐1R receptor inhibitor, not antagonist PLX5622 to remove microglia from the brain (Elmore et al., 2014; Willis et al., 2020). The same Aged SED mice tested above were split into two groups (5–6 mice each), one of which was fed chow containing PLX5622 for 3 weeks, while the other group received standard chow. Histological analysis confirmed that this PLX5622 treatment regimen elicited an >95% reduction in microglia within the hippocampus (Figure 4e,f). This depletion did not alter the number of T‐cells in the aged hippocampus (Figure S8e,f). BAMs, on the other hand, are known to be sensitive to PLX5622 treatment (Willis et al., 2020) and indeed were found depleted from the choroid plexus, meninges and perivascular spaces (Figure S8b–d). Importantly, however, and consistent with our previous findings in young adult control mice (Willis et al., 2020), when re‐testing Aged SED mice in a new APA task (APA2; Figure 4b), we found no impact of PLX5622 treatment on the cognitive performance of Aged SED mice (Figure 4g,h and Figure S8g,h). The number of entries into the shock zone during habituation was again not different here between experimental groups (Aged SED/control chow: 51 ±1 vs. Aged SED/PLX5622: 50 ±5; *p* = 0.87). The combined absence of BAMs and microglia therefore did not influence behavioural outcomes. These findings also suggest that the presence (or state) of either cell type does not actively contribute to the learning deficits of aged mice under basal conditions.

We next examined to what extent microglia contributed to improved cognitive abilities of aged mice under exercise conditions (Figure 4i–l). We assessed three different groups: (1) Aged SED/control mice that were housed under standard conditions and received control chow; (2) Aged RUN/control mice that had access to a running wheel for 21 days and were fed control chow and (3) Aged RUN/PLX5622 mice that had access to a running wheel but were fed PLX5622‐containing chow to induce microglial depletion (Figure 4i). The body weight of aged mice given control chow was not changed by our exercise paradigm (Figure S8i,j). PLX5622 treatment did decrease body weight over the same 21‐day period (Figure S8j), but there was no significant difference in the distance run compared to mice that were fed control chow (Figure S8k). All groups also displayed similar locomotor and/or exploratory behaviour during habituation (Figure 4j) and had a similar number of entries here into the inactive shock zone area (Aged SED/control chow: 104 ± 3 vs. Aged RUN/control chow: 99 ± 3 vs. Aged RUN/PLX5622: 100 ± 4; *p* = 0.46). Both Aged RUN/control and Aged RUN/PLX5622 mice performed significantly better in the APA task (reduction in the number of shock zone entries over time) than Aged SED/control mice (Figure 4k,l and Figure S8l,m). However, there was no significant difference in performance between Aged RUN/control and Aged RUN/PLX5622 mice, demonstrating that the cognitive benefits of exercise in aged mice were conferred irrespective of the presence/absence of microglia. Aged RUN/control and Aged RUN/PLX5622 mice otherwise also had a significantly greater latency to their first entry into the shock zone compared to Aged SED/control mice (Figure S8n); there was again no significant difference between Aged RUN/control and Aged RUN/PLX5622 mice. Taken together, the removal of microglia from the brain did not alter behavioural performance under baseline conditions, nor was it sufficient to block the beneficial effects of exercise on cognitive functioning of aged mice.

At the tissue level, and in line with previous findings, exercise stimulated an increase in pre‐synaptic (Li et al., 2017; Xu et al., 2019) and immature neuronal marker expression (Blackmore et al., 2021; Van Praag et al., 2005), respectively (Figure 4m–q). Compared to Aged SED/control mice, Aged RUN/control mice displayed a significantly greater number of synaptophysin punctae (Figure 4n,o), and also a greater number of immature neurons expressing microtubule‐associated protein doublecortin (DCX; Figure 4p,q; Rao & Shetty, 2004). While the depletion of microglia did not impact synaptophysin results (Figure 4n,o) significantly fewer DCX^pos^ cells were observed in Aged RUN/PLX5622 compared to Aged RUN/control mice (Figure 4p,q). The abundance of TBR2^pos^ intermediate neuronal progenitors was not different between Aged RUN and Aged SED mice in our experimental paradigm (i.e., 3 weeks of running followed by a 2‐week rest period; Figure S8o,p). We otherwise also confirmed that PLX5622 treatment did not alter the numbers of DCX^pos^ immature neurons and/or intermediate neuronal progenitors (TBR2^pos^ cells) under sedentary conditions (Young or Aged; Figure S8q–s).

Together, these findings demonstrate that microglia presence is required for exercise‐mediated neurogenesis in the aged brain. On the other hand, exercise‐induced increases in the expression of synaptophysin, an accepted marker of synaptic plasticity, remained intact following microglia depletion in Aged RUN mice.

## 3 DISCUSSION

Exercise may be useful for preventing (or reversing) age‐related hippocampal deterioration and maintaining neuronal health. However, the mechanisms underlying the beneficial effects of exercise on the ageing brain remain poorly defined. We provide here a comprehensive scRNA‐seq dataset and unbiased analyses characterising the effects of both natural ageing and exercise on cell types within the female mouse hippocampus. We show that ageing alters the relative abundance and transcriptional phenotypes of different cell types in the hippocampus. We further demonstrate that exercise profoundly and specifically impacts the transcriptional state of microglia, reverting the gene expression signature of aged microglia towards that observed in young animals. In particular, the transcriptional profile of disease‐associated microglia was markedly rejuvenated by exercise. We went on to demonstrate that microglia are required for the pro‐neurogenic effects of exercise in the aged hippocampus. Importantly, however, global depletion of microglia did not affect the cognitive benefits conferred by exercise in our experimental paradigm.

### 3.1 Exercise induces a microglia‐specific reversal of biomarkers of ageing

Recent findings from scRNAseq experiments have yielded insights into the transcriptional heterogeneity of microglia in mice and humans on both a temporal and spatial axis, providing new insights into the nature of microglia during development, homeostasis, ageing and disease progression (Allen et al., 2023; Hammond et al., 2018; Masuda et al., 2019). Prior analyses of microglia have indicated that ageing is associated with increased expression of inflammatory factors, including *Lgals3*, *Cst7*, *Ccl4*, *Ccl3 and Il1b*, as well as interferon‐response genes (*Ifitm3* and *Oasl2*) (Allen et al., 2023; Hammond et al., 2019). Here, we identified similar microglial DEGs in association with ageing, including type II interferon and immune genes *Ccl2*, *Ccl3*, *Ccl4*, *Ccl5*, *Ccl7* and *Ccl8*. We also identified pathways enriched in aged microglia, including those regulating the TYROBP causal network, chemokine signalling and type II interferon signalling. Strikingly, the number of microglial DEGs identified between young sedentary mice and aged exercising mice was small, reflecting their transcriptional similarity and hence the restorative impact of exercise on the microglial phenotype. Indeed, our linear regression analyses (Figure 2c and Supplementary note 1 in Data S1) revealed that exercise had a profound and specific effect on the transcriptional signature of aged microglia, reverting their gene expression profile back towards that seen in young microglia. We do acknowledge here that the brains of young adult (3‐month‐old) mice may still go through some complex maturation changes that are separate from the ageing process. Future studies could try to disentangle this through the inclusion of middle‐aged mice, although the rejuvenating effect of exercise on the aged microglial transcriptome already remains undisputed.

Subclustering of the myeloid cell populations present within our dataset defined five distinct types and/or states, each of which was differentially affected by exercise. Of the four bona fide microglial sub‐clusters identified, our linear regression analyses showed that gene expression changes in disease‐associated microglia underpinned much of the observed transcriptional impact of exercise on microglia as a whole (refer to Figure 3d). Closer examination of the DEGs regulated by exercise showed that these included several microglial genes known to be important for plasticity, including neurogenesis and neural circuit formation. For instance, signature genes for ‘disease‐associated microglia’ from the aged hippocampus included *Cst7*, a gene that encodes a cysteine protease inhibitor that limits microglial phagocytosis, and with that, the ability of microglia to support hippocampal neurogenesis (Diaz‐Aparicio et al., 2020; Kang et al., 2018; Sierra et al., 2010).

The rich dataset that we present here paves the way for future studies to better understand the mechanisms by which the genes identified in the different microglial subtypes and/or states contribute to brain ageing and/or health. It should be appreciated, however, that we only used female mice and that the results therefore may not be generalizable to both sexes. Although a comparison to previous work using male mice (Hammond et al., 2019) showed good agreement in terms of the ageing‐related gene expression signature (refer to Figure S6f), future studies would benefit from the inclusion of both sexes to be able to more definitely determine any putative effects of sex (or lack thereof) on the ageing and exercise response.

### 3.2 The transcriptional profile of hippocampal BAMs is mostly unaffected by exercise

Our subclustering analysis also identified a population of BAMs, which are CNS‐associated macrophages residing in the perivascular spaces and pial folds of the hippocampus; these cells are anatomically, developmentally and transcriptionally distinct from bona fide microglia (Utz et al., 2020). Much remains to be discovered about the function of BAMs under pathological conditions, but several lines of evidence support their involvement in the progression of brain diseases in ways that are distinct from microglia (Kierdorf et al., 2019); novel roles in CSF flow dynamics have also recently been uncovered (Drieu et al., 2022), which may impact on immune regulation (Piehl et al., 2022). Here we found that the proportion of BAMs in the hippocampus was markedly increased in aged mice, but exercise reversed this change. Intriguingly, however, and in contrast to microglial sub‐clusters, the transcriptional profile of BAMs remained largely unaffected by exercise. Removal of BAMs from our linear correlation analyses further confirmed that BAMs did not drive the exercise‐induced changes in the transcriptional profile of resident microglia/macrophages in the aged mouse brain. This differential response of BAMs compared to other brain myeloid cell populations is consistent with findings in other contexts, including experimental autoimmune encephalomyelitis (Jordao et al., 2019) and Alzheimer's disease (Mrdjen et al., 2018). The mechanisms and interactions underlying population size and cellular responses of BAMs to ageing and exercise are now topics for further investigation.

### 3.3 Microglia mediate the pro‐neurogenic effects of exercise in the aged hippocampus

Recent work from our group highlighted that microglial phenotypes can profoundly influence hippocampal neurogenesis in the injured brain (Willis et al., 2020). The process of adult neurogenesis itself is otherwise also well known to be regulated and/or influenced by exercise. We previously demonstrated that exercise supports both the activity and survival of neural precursors (Blackmore et al., 2021; Vukovic et al., 2012), and that microglia may play a role therein (Vukovic et al., 2012). With our unbiased single‐cell transcriptomics analyses identifying microglia as the cells mostly modulated by exercise, we probed the in vivo significance of this phenomenon in relation to hippocampal neurogenesis. Here, our depletion experiments showed that the loss of microglia annulled any stimulatory effects of exercise on hippocampal neurogenesis. As technological advances progress, future studies could explore more specifically what microglial subset (or state) inhibits adult neurogenesis and/or drives the pro‐neurogenic effects of exercise.

### 3.4 Benefits of exercise on cognition are retained in absence of microglia

Our study provides insights into the contribution of microglia towards ageing‐associated cognitive decline. Age‐related deficits have been reported for microglia, gradually impeding their ability to perform vital homeostatic functions (Conde & Streit, 2006). However, there is still uncertainty as to whether microglia in the aged hippocampus transition from a homeostatic state to an enhanced pro‐inflammatory phenotype that contributes to neuronal senescence and cognitive decline and/or whether aged microglia simply possess a dysfunctional response to CNS perturbations.

In support of the former, several studies have suggested that the enhanced pro‐inflammatory state of microglia in the aged brain may actively contribute towards age‐related deficits in cognition and memory (Barrientos et al., 2006; Jurgens & Johnson, 2012; Ojo et al., 2015; Olah et al., 2018). An intrinsic pro‐inflammatory microglial phenotype in the aged brain has been proposed based on their enhanced expression of inflammatory factors in response to lipopolysaccharide (LPS) challenge (Elmore et al., 2018; Niraula et al., 2017; O'Neil et al., 2018). The changing microenvironment of the ageing brain itself has also been implicated in the conferral of a maldaptive microglial phenotype, a premise supported by studies in which newborn microglia in the aged brain (achieved via genetically‐induced microglial turnover and replenishment) still exhibited a heightened inflammatory response to LPS challenge (O'Neil et al., 2018). Here, we more directly probed the role of aged hippocampal microglia on cognition by removing these cells with the brain‐penetrant CSF‐1R inhibitor PLX5662. We observed no differences in APA performance of aged mice with and without microglia. These findings suggest that, despite their primed state (O'Neil et al., 2018), the phenotype and/or mere presence of microglia in the aged brain did not actively influence performance in the cognitively challenging APA task. This finding is consistent with our prior study, in which we showed that pharmacological removal of microglia does not interfere with a range of spatial learning and memory outcomes in young adult mice under either sham‐operated or traumatic brain injury conditions (Willis et al., 2020). This is not to say, however, that cumulative microglia dysfunction with ageing does not precipitate in and/or contribute to brain pathology, and it would be of interest to examine this further in follow‐up studies.

The finding that the benefits of voluntary wheel running in relation to the cognitive abilities of aged mice were retained with microglia depletion was unexpected. In our experiments, the wider impacts of exercise on the brain and/or body physiology as a whole would appear to be more key therefore in terms of conferring the observed cognitive improvements in aged mice. An inherent limitation of brain scRNA‐seq is the very limited presence and/or absence of neurons within the dataset, and future studies could adopt single‐nucleus RNAseq to also consider exercise effects on the neuronal transcriptome in the aged brain. Changes in neuronal function and/or plasticity were already evident from synaptophysin staining (a reliable and accepted indicator of synaptic plasticity), providing clues for at least one alternative mechanism via which the benefits of exercise may be conferred. We have otherwise also identified another cell type of interest (i.e. T cells) whose abundance was modified by exercise, not just in the aged hippocampus but also peripherally in the liver. The latter finding is in agreement with a recent study demonstrating that exercise can rescue ageing‐associated increases in cytotoxic and memory CD8^pos^ T cell numbers across a range of tissues, including peripheral sites such as the bone marrow, lung and liver (Sun et al., 2023). With ageing, we identified T cell‐attracting chemokines like *Ccl5* being expressed by both microglia and astrocytes, and it would therefore be of interest to further explore the role of these adaptive immune cells in relation to essential processes underlying the integrity and/or function of neural circuits that underpin spatial learning and memory. Lastly, we do acknowledge that only one behavioural paradigm (i.e., APA acquisition) was used in the present study. That said, our previous work showed that the beneficial effect of exercise on attenuating the ageing‐related cognitive impairments is not confined to this robust task and can be recapitulated in the Barnes maze (Blackmore et al., 2021). Future studies could address whether microglia depletion may yield more subtle effects on other behaviours not tested in the APA task.

### 3.5 Cellular composition of the hippocampus is altered with ageing and exercise

Several recent RNA sequencing studies have used either whole brain or individually isolated cell types to investigate the impact of ageing on gene expression profiles, identifying region‐ and cell‐type‐specific effects (Allen et al., 2023; Hammond et al., 2019). Here, we closely examined nine different cell types from the hippocampus of young and aged mice through scRNAseq, with the addition of voluntary exercise as an interventional variable. In agreement with previous reports (Allen et al., 2023; Altendorfer et al., 2022; Kaya et al., 2022), and as alluded to earlier, we found that natural ageing was associated with significant T cell accumulation in the brain. Strikingly, we further discovered that this hallmark of brain ageing was reversible, with exercise having a strong restorative effect in that it led to a marked reduction in T cell presence within the hippocampus. It is tempting to speculate that a reduction in the production of various T cell‐attracting chemokines from ageing microglia (and/or astrocytes), discussed above, may drive this effect of exercise (Zhang et al., 2022).

Future studies can now explore this further, including a putative role of (endothelial) Ackr1 therein (Mapunda et al., 2022) and also the link between T cell presence and cognitive (dys)function (Groh et al., 2021). An alternative could be to investigate whether exercise changes the molecular microenvironment away from one that might locally support T cell maintenance and/or activation in the ageing brain. It would similarly be of interest to examine the extent to which any exercise‐induced reductions in T cell presence are a factor of influence in the rejuvenation of microglia or, alternatively, whether T cells contribute to the transcriptional signature of aged hippocampal microglia.

## 4 CONCLUSIONS

We identify microglia as a key cellular target of exercise, with this intervention attenuating and/or counteracting age‐related changes. We also highlight a requirement for the presence of rejuvenated microglia in relation to exercise‐induced neurogenesis in the hippocampus of aged mice. Overall, the transcriptional signatures presented here for microglia and other cell types in terms of ageing and/or their responsiveness to exercise provide a rich resource for further functional interrogation that should help (re)define how cell states contribute to cognitive decline and/or the reversal thereof.

## 5 EXPERIMENTAL PROCEDURES

### 5.1 Animals

Female C57BL/6 mice (Animal Resource Centre, Australia) were used unless otherwise stated. All ‘aged’ mice were 18 months old at the beginning of the experiment; ‘young’ mice were 3 months old. All mice were housed socially throughout the experiments in groups of 2–5 animals per cage and maintained on a 12‐hour light/dark cycle with food and water provided ad libitum. All experiments were conducted in accordance with the Australian Code for the Care and Use of Animals for Scientific Purposes, with approval from The University of Queensland's Animal Ethics Committee.

### 5.2 Exercise paradigm

Mice were housed in groups of 2–3 mice in wire cages (51 × 22 × 13 cm) with a free‐moving running wheel suspended from the top. Mice in the exercise treatment group (“RUN”) had unrestricted access to the running wheel during the exercise paradigm (21 days exercise, followed by a 14‐day rest period), whereas sedentary (“SED”) mice did not have access to a running wheel. All mice were provided with Pura Chip Aspen Fine Sani Chips and Enviro‐Dri as their bedding and tissue for standard enrichment purposes. Mouse body weight was recorded at the beginning (day 0) and end (day 21) of the running period. Voluntary wheel running was monitored by an odometer and assessed daily across the 21‐day running period; the mean running distance was calculated by dividing the total distance per day per number of mice in each cage.

### 5.3 PLX5622 treatment

To deplete microglia, mice were provided ad libitum with chow containing 1200 ppm PLX5622 (Plexxikon, USA; used under the permission of a material transfer agreement). Control mice were fed standard chow (AIN‐76A, Research Diets). See the experimental timelines in each figure for the timing of PLX5622 administration.

### 5.4 Sample preparation for single cell RNA‐seq

Hippocampi (*n* = 3 mice per group/condition) were isolated from young sedentary (“Young SED”), aged sedentary (“Aged SED”) and aged runner (“Aged RUN”) mice, as previously described (Willis et al., 2020; Willis & Vukovic, 2020). We carefully considered possible sources of variation and also technical confounders here. To this end, three littermates (one per condition) were processed in parallel, with experiments repeated over three separate days. Cells were also kept on ice wherever possible to slow metabolic processes, including transcription. Overall, mice were euthanized using an intraperitoneal (i.p.) injection of sodium pentobarbitone (1.6 mg/g BW) before being transcardially perfused with 15 mL phosphate buffered saline (PBS). Brains were immediately removed, the hippocampi isolated and diced with a scalpel. Hippocampal tissue was digested for 16 min at 37°C in 0.1% papain (Worthington Biochemical Corporation) and 0.1% DNaseI (Roche Australia) in HBSS (Thermo Scientific), with titration to dissociate the tissue. Cells were washed and resuspended in wash media (HBSS containing 10% foetal calf serum), then filtered into a single cell suspension through a 100‐μm sieve (Falcon, BD Biosciences). Dissociated single cells were then sorted by fluorescence‐activated cell sorting (FACS, BD Influx Cell Sorter) into PBS with 10% foetal calf serum (FCS) for single cell sequencing detailed below.

### 5.5 Single cell RNA‐seq library preparation

High‐throughput droplet partitioning of viable cells with barcoded beads was performed using the 10X Genomics Chromium instrument (10X Genomics) and the Single Cell 3′ library, Gel Bead and Multiplex Kit (v1; 10X Genomics; PN‐120237). The number of cells loaded into each reaction was optimized to capture approximately 5000 cells. cDNA was prepared from the resulting partitioned samples. cDNA shearing was performed with a Covaris S2 instrument (Covaris) set to produce a target size of 200 bp as per manufacturer's recommendation (intensity: 5, duty cycle: 10%; cycles: 200; time: 120 s). The resulting single‐cell transcriptome libraries were pooled and sequenced on an Illumina NextSeq500 using a 150‐cycle High Output reagent kit (NextSeq500/550 v2; Illumina, FC‐404‐2002) in standalone mode as follows: 98 bp (Read 1), 14 bp (I7 Index), 8 bp (I5 Index) and 10 bp (Read 2).

### 5.6 Sequencing reads alignments

10X genomics Cell Ranger software (version 3) was used to process the raw base call files (BCL) obtained from sequencing. Briefly, BCL files were demultiplexed and converted into FASTQ files using the *mkfastq* function. Demultiplexed reads were aligned to the GRCm38.p6 mouse reference genome, cell barcodes were identified, and unique molecular identifiers (UMI) were counted using the *count* function. The results were compiled in a matrix with columns relating to cells and rows relating to genes. Cells from empty droplets were removed using the emptyDrops function in the DropletUtils R package (Lun et al., 2019).

### 5.7 Quality control

Healthy cells were distinguished from damaged cells by three quality control metrics (Figure S1a–d): (1) percentage of mitochondrial genes; (2) number of unique genes per cell and (3) number of UMIs per cell. The percentage of mitochondrial genes acts as a readout for cell health here due to the pivotal roles of mitochondria in controlling apoptosis. In agreement with the literature (Mercer et al., 2011), hippocampal cells were selected as ‘healthy’ if less than 15% of their total expressed genes were mapped to the mitochondrial genome. To remove cell‐ and gene‐outliers, a threshold of three standard deviations (SD) was applied from the mean number of unique genes per cell and the mean number of UMIs per cell.

### 5.8 Cell clustering

Identified healthy cells were clustered together using the R software package Seurat (version 3). Briefly, gene counts were normalised based on library size. UMIs were normalised across cells, scaled per 10,000 and converted to log scale using the ‘NormalizeData’ function. Normalised counts were then converted to z‐scores using the ‘ScaleData’ function. Highly variable genes were selected with the ‘FindVariableGenes’. Two thousand variable genes were then used to calculate principal components with the ‘RunPCA’ function. Euclidean distance between cells was calculated with the ‘FindNeighbors’ command and used to identify clusters (using ‘FindCluster’ with a resolution_parameter = 1.0, the Leiden algorithm and the first 40 principal components). Finally, to visualise the data, a Uniform Manifold Approximation and Projection (UMAP) was calculated from the first 40 principal components.

### 5.9 Cell annotation

UMAP clusters were identified using an array of cell type markers reported in the literature. Using the following markers, eight cell populations were identified: astrocytes (*Slc1a2*, *Aldoc*, *Slc1a3*, *Aqp4; n = 2240*), endothelial cells (*Tek*, *Cldn5*, *Cdh5*, *Kdr; n = 1504*), microglia (*Cx3cr1*, *Tmem119*, *Aif1*, *Cd68; n = 1478*), neuronal precursors (*Meg3*, *Nhlh2*, *Rtn1*, *Gap43; n = 197*), oligodendrocytes (*Mbp*, *Sox10*, *Olig1*, *Mog; n = 2803*), oligodendrocyte precursors (*Pdgfra*, *Olig2*, *Cspg4*, *Ptgds; n = 303*), pericytes (*Cd248*, *Kcnj8*, *Pdgfrb*, *Abcc9; n = 387*) and T cells (*Trbc2*, *Trac*, *Cd3d*, *Cd3g; n = 197*). In addition, there was a population of cells that we were unable to identify based on their gene expression signatures (*n* = 129). References for papers used to identify these cell‐type markers are provided in Table S1.

### 5.10 Differential gene expression analyses

Differentially expressed genes (DEGs) were identified between experimental groups (Young SED vs. Aged SED and Aged SED vs. Aged RUN) using the Seurat ‘FindMarkers’ function for genes showing any fold‐difference (in log‐scale) between two experimental conditions (Wilcoxon Rank Sum test). A threshold of 0.05 was used after Bonferroni correction for the number of genes tested in each cell types. *p*‐values were corrected for 15,307 tests in microglia, 14,547 in endothelial cells, 16,488 in oligodendrocytes and 16,773 in astrocytes.

### 5.11 Pathway enrichment analysis

Functional enrichment analysis was performed from differentially expressed genes (DEGs) identified between conditions. To obtain a list of enriched pathways, ‘gost’ from the gprofiler2 R package was used, with ‘function’ parameters set according to the WIKIPATHWAY database. The WIKIPATHWAY database (August 2022 release) was selected due to its ease of biological interpretation compared to other pathway databases. Briefly, the gost function perform enrichment analysis using a hypergeometric test to investigate whether a list of DEGs identified is found more often than would be expected at random. To perform the enrichment, the DEGs are compared to a background gene list (i.e. all the genes that can potentially be found). This was set as the genes found in each individual cell type. The *p*‐value obtained from the hypergeometric test was then corrected for the number of pathways investigated using the g:SCS threshold method (Raudvere et al., 2019).

### 5.12 Cell type proportion test

The proportions of each cell type present in each experimental group were compared using a Monte‐Carlo simulation and a permutation test. Briefly, our null hypothesis was that there were no differences in the proportions of cell types observed between conditions. To test this hypothesis, cells were pooled and randomly segregated into their original library size 10,000 times. The differences in the cell type proportions were recalculated from the pooled cells. The *p*‐value was calculated from the number of simulations showing similar proportional differences over all simulations. To avoid false positives, a doubling of proportion between experimental groups was set as our threshold for significance. This approach is implemented in the scProportionTest R package.

### 5.13 Linear regression analysis

To understand the overall effect of exercise on markers of ageing, we compared the log foldchange between our Young SED and Aged SED mice (LFC_YngSED_AgSED) and Young SED and Aged RUN mice (LFC_YngSED_AgRUN), using linear regression performed via the following call in R: ‘lm(LFC_YngSED_AgRUN ~ LFC_YngSED_AgSED)’. Here, a regression coefficient (R^2^) close to 1 implies that the ageing‐related gene expression changes in Aged SED and Aged RUN compared to Young SED are similar, i.e., indicating little effect of exercise on these genes. On the other hand, an R^2^ of zero indicates that exercise completely reverses ageing‐related gene expression.

To externally validate and/or determine the robustness of ageing effects within our dataset, we also compared genes that were differentially expressed between microglia from Young SED and Aged SED mice with the dataset from Hammond et al. (2019), which also characterised gene expression changes in microglia in mice during development and ageing. Individual libraries for the relevant timepoints were first subjected to quality control measures similar to our own and then merged (Figure S6a–d). Following this integration, markers of ageing were calculated between young and aged mice. To assess the correlation between our microglial ‘ageing signatures’ and that of Hammond et al. (2019), we again performed linear regression analysis between the log fold change from the Young SED and Aged SED comparison (LFC_YngSED_AgSED) and the log fold change between young and aged mice in the Hammond dataset (LFC_Y_Ag_Hmd) using the following function in R: ‘lm(LFC_YngSED_AgSED ~ LFC_Y_Ag_Hmd)’.

### 5.14 Downsampling analysis

To account for the potential effect of an unbalanced number of cells captured within experimental groups, downsampling was performed by randomly removing cells from the most numerous groups to match the smaller ones. Differentially expressed genes were identified as per the previous comparisons (Young SED vs. Aged SED and Aged SED vs. Aged RUN), and Pearson correlation was used to compare the log foldchange obtained before and after downsampling. Finally, downsampled log foldchanges were also used to perform linear regression as previously described.

### 5.15 RNA velocity analysis

Microglia were subsetted and read into Python using scanpy (Wolf et al., 2018). The Python package scVelo (La Manno et al., 2018) was then employed to perform RNA velocity quantification in microglia only using default setting. Matplotlib was used to plot the UMAP reduction calculated previously in R.

### 5.16 Cell–cell interaction analysis

To perform cell–cell interaction, we accessed CellPhoneDB (Efremova et al., 2020), a repository of ligand receptor interactions through the R package CellChat (Jin et al., 2021). To identify significant interactions, we performed random permutation of pairwise cluster labels and obtained the average expression of ligand and receptors over those clusters. Comparing the expression obtained in our dataset to the created null distribution allowed us to identify significant ligand‐receptor interactions. Only genes identified as differentially expressed between Young SED and Aged SED or Young SED and Aged RUN mice were included in the analysis to identify putative changes in ligand‐receptor interactions within our experimental design.

### 5.17 Plotting of RNAseq data

UMAP plots were obtained using scater (McCarthy et al., 2017), barplots using dittoSeq (Bunis et al., 2020), heatmaps using Seurat (Stuart et al., 2019) and dot plots using ggplot2.

### 5.18 Active place avoidance

To assess spatial learning and memory, mice were tested on the active place avoidance (APA) task, as previously described (Vukovic et al., 2013; Willis et al., 2017). In this task, mice are placed on a moving turntable and must use visual cues and adjust their position accordingly to avoid entering a stationary shock zone. The APA arena consisted of a rotating (1 rpm) elevated platform with a grid metal floor, fenced by a 32 cm‐high clear Perspex boundary, with distinct black and white A3‐sized visual cues placed on the four surrounding walls. An unmarked shock zone (the position of which stayed constant in relation to the room) occupied 60° of the circular arena.

In the 7 days prior to behavioural testing, mice were habituated to the experimenter by being placed on the experimenter's arm for 1 min/day. One day before testing, mice were habituated to the rotating APA arena by being allowed to freely explore the space with the shock zone turned off. For the exercise experiment in aged mice (Figure 4i–l), mice were allowed 20‐min trials. For other cohorts (e.g., young vs. aged; Figure 4a–h), mice were allowed 10‐min trials. Daily tests were conducted over five consecutive days. Mice were always placed into the arena directly opposite the shock zone location. Mice were tracked by an overhead camera linked to Tracker Analysis software (Bio‐Signal Group), with entry into the shock zone leading to the delivery of a brief foot shock (0.5 mA, 500 ms duration, 60 Hz, at 1.5 s intervals). Spatial learning and memory were assessed by analysing the number of entries into the shock zone, latency to first entry and the maximum duration of shock zone avoidance per day using the Track Analysis software (Bio‐Signal Group).

For experiments involving the re‐testing of mice, baseline measurements of spatial learning and memory (APA1) were assessed for each mouse by calculating the percentage improvement between the number of shock zone entries observed on day one compared to day five. Next, mice were assigned to experimental treatment groups such that each group had an approximately equal mean percentage improvement in the baseline measurements. After experimental treatment, mice were given another APA test (APA2) to assess spatial learning and memory. To avoid confounding effects (i.e. mice remembering the previous paradigm), visual cues were changed, the rotation direction of the arena reversed, and the position of the shock zone moved 180° compared to the conditions in APA1.

### 5.19 Tissue preparation for immunohistochemistry

Mice were euthanized using an i.p. injection of sodium pentobarbital (1.6 g/kg; Verbac) before being transcardially perfused with ice‐cold phosphate buffered saline (PBS) and 10% formalin (Sigma). Brains and peripheral tissues (i.e. liver) were dissected and then placed in 10% formalin for 24 h to fix tissue. Tissues were then transferred into PBS with 0.01% (w/v) sodium azide (Sigma) and stored in the dark at 4°C.

Prior to sectioning, brains were cryoprotected in 30% (w/v) sucrose in PBS with 0.01% (w/v) sodium azide for 2 days at 4°C or 24 h at room temperature. Serial 1‐in‐6 coronal brain sections (40 μm) were collected using a sliding microtome (Leica) and stored free‐floating in PBS with 0.01% (w/v) sodium azide at 4°C.

Liver samples were dehydrated in increasing concentrations of ethanol, followed by clearing in xylene and embedded in paraffin. Tissues were sectioned into 5 μm sections using a rotary microtome (ThermoFisher) and mounted onto superfrost microscope slides for staining.

### 5.20 Immunohistochemistry

For the study of adult neurogenesis, microglia presence and/or depletion, brain sections were blocked in 5% (v/v) normal goat serum (NGS) with 0.3% (v/v) Triton‐X 100 (Sigma) in PBS before being incubated overnight at 4°C with primary antibodies: rabbit anti‐IBA1 (1:1000; Wako), guinea pig anti‐DCX (1:1000; Millipore) diluted in 3% (v/v) NGS with 0.1% (v/v) Triton‐X 100 in PBS. Sections were washed in PBS three times for 5 min before being incubated with secondary antibodies: Alexa Fluor goat anti‐rabbit 488 (1:2000, Thermo Fisher) and Alexa Fluor goat anti‐guinea pig 488 (1:2000, Thermo Fisher) diluted in 3% (v/v) NGS with 0.1% (v/v) Triton‐X 100 in PBS.

For in vivo validation of select DEGs, free floating serial brain sections were first photobleached for at least 24 h. Antigen retrieval was performed using sodium citrate (10 mM, pH 6.0) with 0.05% Tween‐20 at 95°C for 15 min, before being rapidly cooled on ice and washed three times with 1xPBS. Brain sections were blocked in 5% bovine serum (BSA, v/v) with 0.3% Triton‐X 100 (Sigma) in PBS before being incubated overnight at 4°C with primary antibodies: goat anti‐CD31 (1:1000, AF3628, R&D Systems), rabbit anti‐SPP1 (1:500, ab8448, Abcam), rabbit anti‐CD74 (1:500, ab245692), goat anti‐IBA1 (1:1000, ab5076, Abcam), mouse anti‐6E10 (1:400, cat#803001, Biolegend), rabbit anti‐synaptophysin (1:500, ab32127, Abcam), rat anti‐CD8a (4SM15, 1:500, cat# 14–0808‐37, eBioscience), rat anti‐CD3 (1:500, ab11089, Abcam) and/or rat anti‐CD4 (1:400, BD Bioscience, cat# 553927), rat anti‐CD11b (1:500, MA1‐80091, Invitrogen) diluted in 3% (v/v) BSA with 0.1% (v/v) Triton‐X 100 in PBS. Sections were washed in PBS three times for 10 minutes before being incubated with secondary antibodies for 2 h at room temperature: Alexa Fluor 594 AffiniPure donkey anti‐rat IgG (H + L, 1:1000), Alexa Fluor 594 AffiniPure donkey anti‐rabbit IgG (H + L, 1:1000), Alexa Fluor 647 AffiniPure donkey anti‐mouse IgG (H + L, 1:1000), Alexa Fluor 488 AffiniPure donkey anti‐goat IgG (H + L, 1:1000), Alexa Fluor 647 AffiniPure donkey anti‐rabbit IgG (H + L, 1:1000) diluted in 3% (v/v) BSA with 0.1% (v/v) Triton‐X 100 in PBS. Primary antibody omissions were used to confirm staining specificity.

All stained brain sections were washed in PBS three times for 5 min after secondary antibody incubation, with the nuclear stain 4′,6‐diamidino‐2‐phenylindole dihydrochloride (DAPI, 1:1000) added in the last wash. Next, sections were mounted onto glass slides (Menzel Glaser), immersed with Vectashield H‐100 mounting medium (Vector Labs) and then coverslipped (Menzel Glaser) for analysis.

For liver, paraffin‐embedded sections were deparaffinized in xylene, followed by sequential rehydration in ethanol and immunostained as previously described (Shaikh et al., 2023). Sections were blocked in 5% bovine serum (BSA, v/v) with 0.3% Triton‐X 100 (Sigma) in PBS before being incubated overnight at 4°C with primary antibodies: rat anti‐CD3 (1:500; ab11089, Abcam). Sections were washed three times in PBS for 10 min each and then incubated with secondary antibodies for 2 h at room temperature (Alexa Fluor 647 AffiniPure donkey anti‐rat IgG (H + L, 1:1000), diluted in 3% BSA with 0.1% (v/v) Triton‐X 100 in PBS). Sections were again washed with PBS, stained with DAPI, mounted and coverslipped as described above.

### 5.21 Imaging and cell quantification

For quantification of DCX^pos^ cells or CD3^pos^ T cells, five consecutive sections of the dorsal dentate gyrus starting at approximately anterior–posterior (AP) −1.5 mm from Bregma were quantified per mouse. Cells were counted on a fluorescent stereology microscope (Carl Ziess), using a 40× 0.75 NA air objective and running Stereo Investigator software (v2017.01.1, MBF Bioscience). DCX^pos^ cells counts were normalised to the length of the subgranular zone (SGZ) in each section. T‐cells (CD3^pos^, CD4^pos^ or CD8a^pos^) were normalised to the analysed area of the hippocampus (e.g., dentate gyrus, hilus, CA1, CA3, whole hippocampus), and the mean cell count across five sections calculated for each mouse. T‐cells were considered perivascular when associated with CD31^pos^ structures. IBA1‐labelled BAMs in the meninges, choroid plexus and perivasculature were quantified as described previously (Willis et al., 2020).

For quantification of IBA1^pos^ microglia, five consecutive sections starting at approximately AP −1.5 mm were imaged on a spinning‐disk confocal microscope (3i Instruments with Yokogawa W1 spinning disk module), using a 20× 1.2 NA air objective, a Hamamatsu Flash 4.0 sCMOS camera and running SlideBook software (v6.0.16, 3i Instruments). For quantification of microglia in the dentate gyrus, a 16‐bit 3D montage of the full 40 μm physical depth of the section was acquired with a z‐interval of 1.2 μm and compressed into a maximum z‐projection. Cell counts were quantified using FIJI software by cropping a region of interest (ROI) around the dentate gyrus and manually counting cells within the ROI or field of view using the “Cell Counter” plugin. Cell counts were normalised to the 2D area of the ROI or field of view, and the mean cell count across five sections was calculated for each mouse.

Analysis of CD74 and SPP1 mean fluorescent intensity (MFI) in IBA1^pos^ or CD11b^pos^ microglia in the dentate gyrus of the hippocampus was performed as previously described (Harley et al., 2021; Willis et al., 2020). Confocal images were acquired for the hippocampus using an inverted Diskovery spinning disk confocal microscope (Spectral Applied Research) with a Zyla sCMOS camera at 20× magnification using a CFI Plan Aprochromat VC 20x/N.A. 0.75/W.D. 1.0 mm objective. Imaris software was used to assess MFI, calculated as the mean from four consecutive sections at Bregma: −1.50 mm, −1.70 mm, −1.94 mm, −2.18 mm, per mouse. All sections were stained in one batch with the same imaging threshold and exposure time to ensure consistency for image analysis.

For quantification of synaptophysin (SYN) puncta, confocal images of the hippocampus were acquired using an inverted Diskovery spinning disk confocal microscope (Spectral Applied Research) with a Zyla sCMOS camera at 60× magnification using a CFI Apo Lambda 60× Oil/N.A. 1.4/W.D. 0.13 mm objective. Imaris software was used to quantify the number of SYN puncta in the dentate gyrus per analysed volume of the tissue section. Quantification was performed blinded and calculated as the mean from four consecutive sections at Bregma: −1.50 mm, −1.70 mm, −1.94 mm and −2.18 mm, respectively, per mouse.

### 5.22 Statistics

Statistical analyses were conducted using GraphPad Prism software (version 7.02). For analysis using an unpaired, two‐tailed Student's *t*‐test, a Welch's correction was used if the standard deviation of groups differed by >2×. For analysis using either one‐ or two‐way analysis of variance (ANOVA), a Greenhouse–Geisser correction and Bonferroni post‐hoc comparison were used. Values are given as mean ± SEM, with replicates representing individual animals unless specified otherwise. Statistical significance was set at *p* < 0.05 after correction for experiment‐wise multiple testing.

## AUTHOR CONTRIBUTIONS

Conceptualization, J.V.; Methodology, S.C., J.V., M.J.R., E.F.W., S.H., S.S., A.S., J.P., N.W., Q.N.; Investigation, S.C., E.F.W., M.J.R., S.H., J.V.; Writing – Original Draft, E.F.W. and J.V.; Writing – Review & Editing, S.C., S.S., J.V., E.F.W. and M.J.R; Funding Acquisition, J.V.; Resources, J.V.; Supervision, J.V., S.S. and Funding, J.V.

## CONFLICT OF INTEREST STATEMENT

The authors declare no competing interests.

## Supporting information


Figure S1.

Figure S2.

Figure S3.

Figure S4.

Figure S5.

Figure S6.

Figure S7.

Figure S8.

Data S1.

## Data Availability

The scRNA‐seq data generated in this study is available from figshare [https://doi.org/10.6084/m9.figshare.21756086]. Data from Hammond et al. (2019) was obtained from the genome omnibus repository at the following ascension number: GSE121654. The code used to generate this study data can be found on github: https://github.com/CNSGenomics/SingleCell‐MicrogliaAging.
