# Supplementary figures and images for: Exercise rejuvenates microglia and reverses T cell accumulation in the aged female mouse brain
Source: Aging Cell. 2024 May 15;23(7):e14172. doi: 10.1111/acel.14172 (PMC11258432; doi:10.1111/acel.14172)

Supplementary Figure 1 (Information for figure 1)

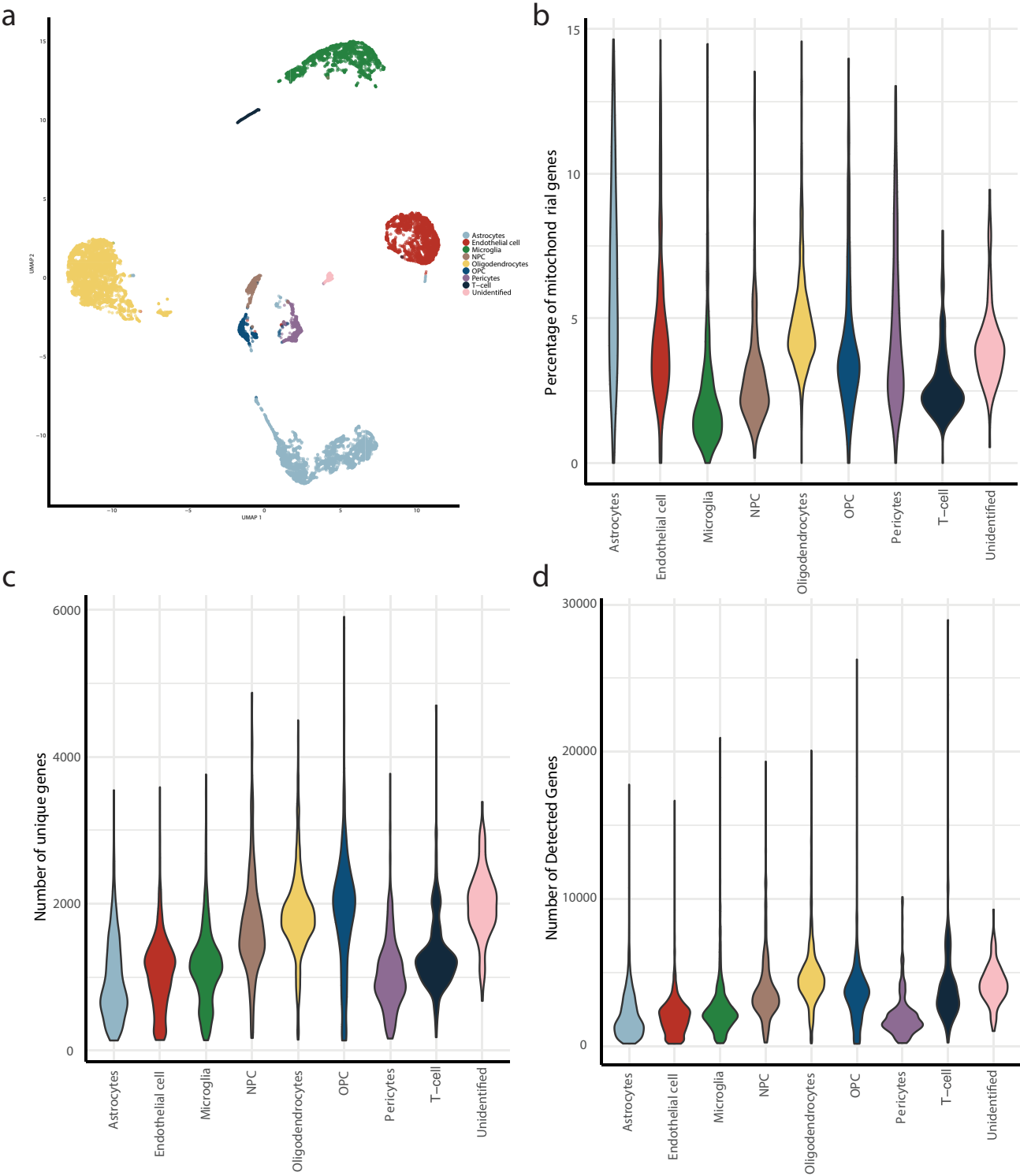

Supplement: Supplementary file 1 — Figure S1. [file ACEL-23-e14172-s005.pdf]

Supplementary Figure 2 (Information for figure 1)

a

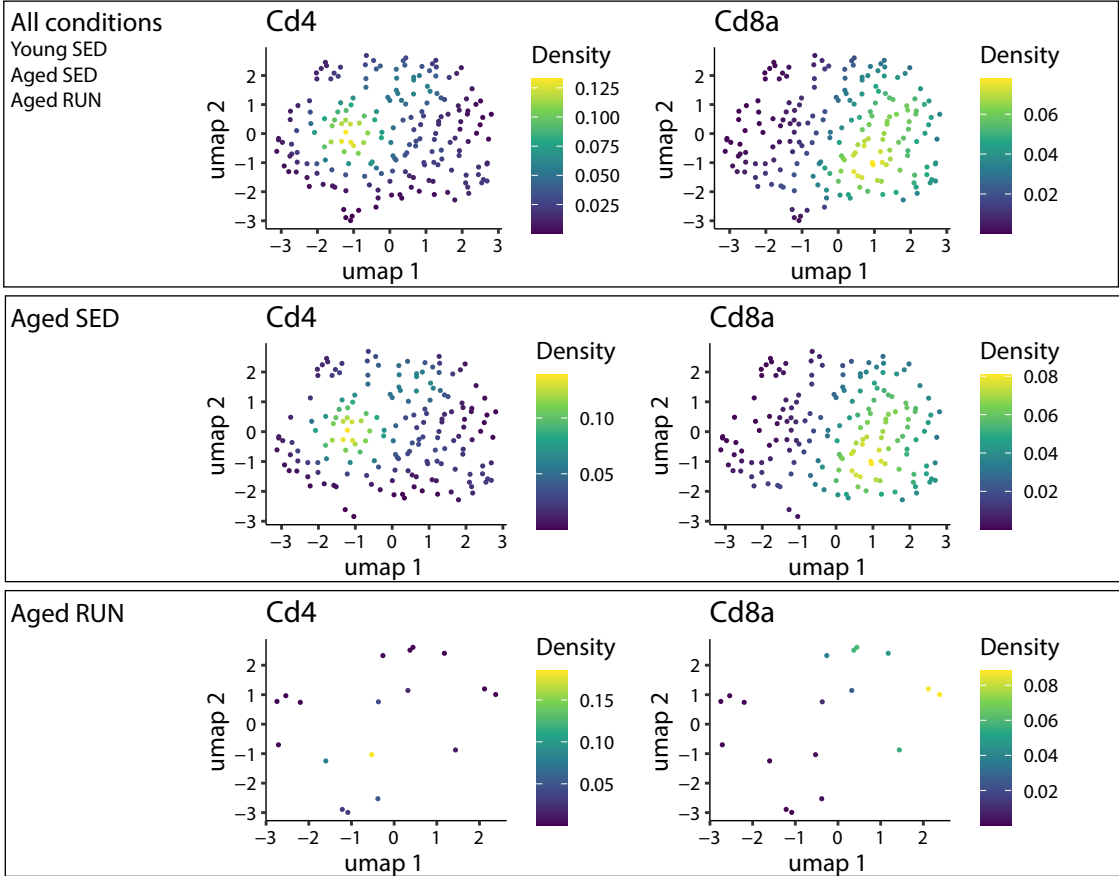

b

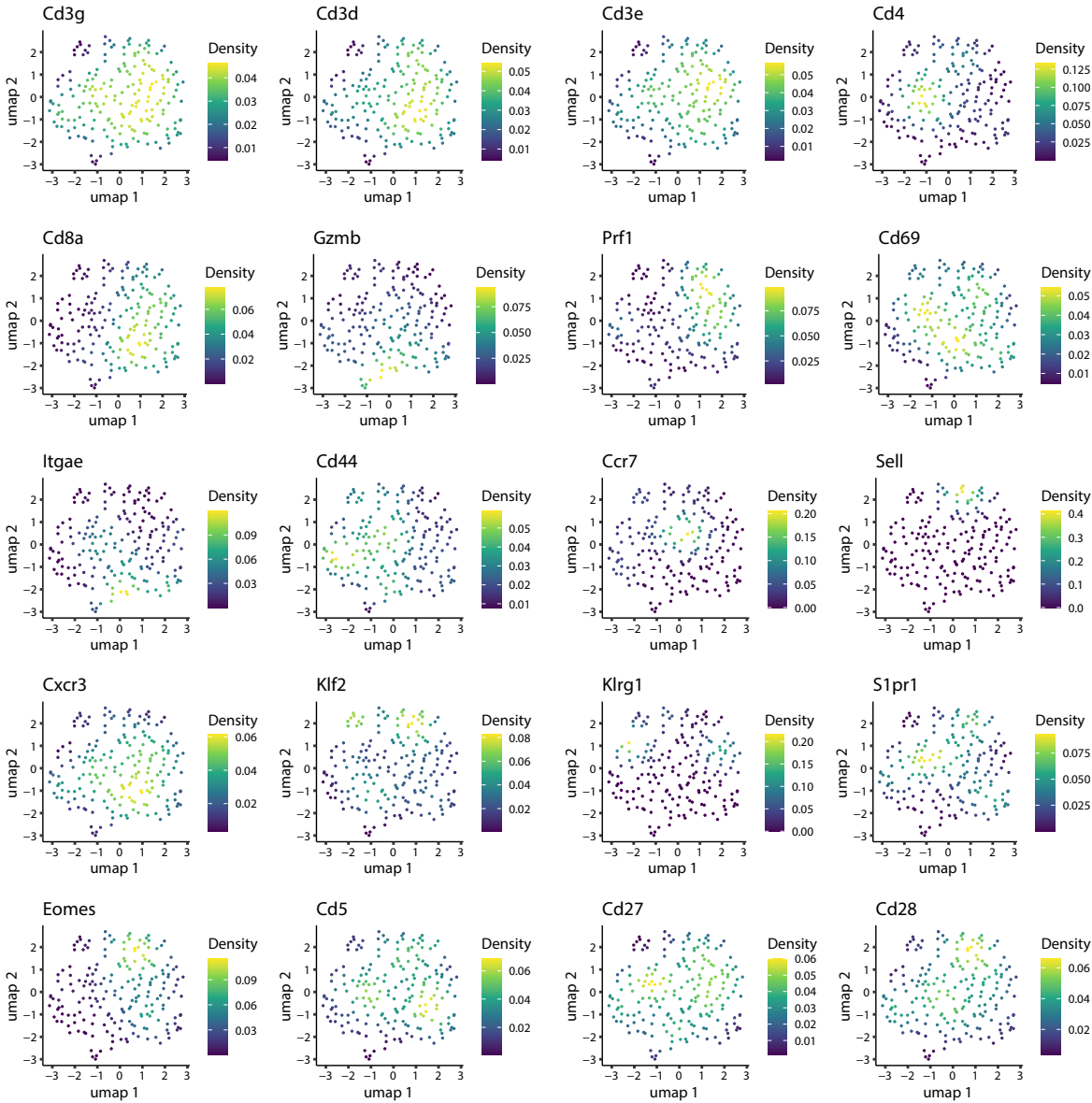

Supplement: Supplementary file 2 — Figure S2. [file ACEL-23-e14172-s004.pdf]

Supplementary Figure 3 (Information for figure 1)

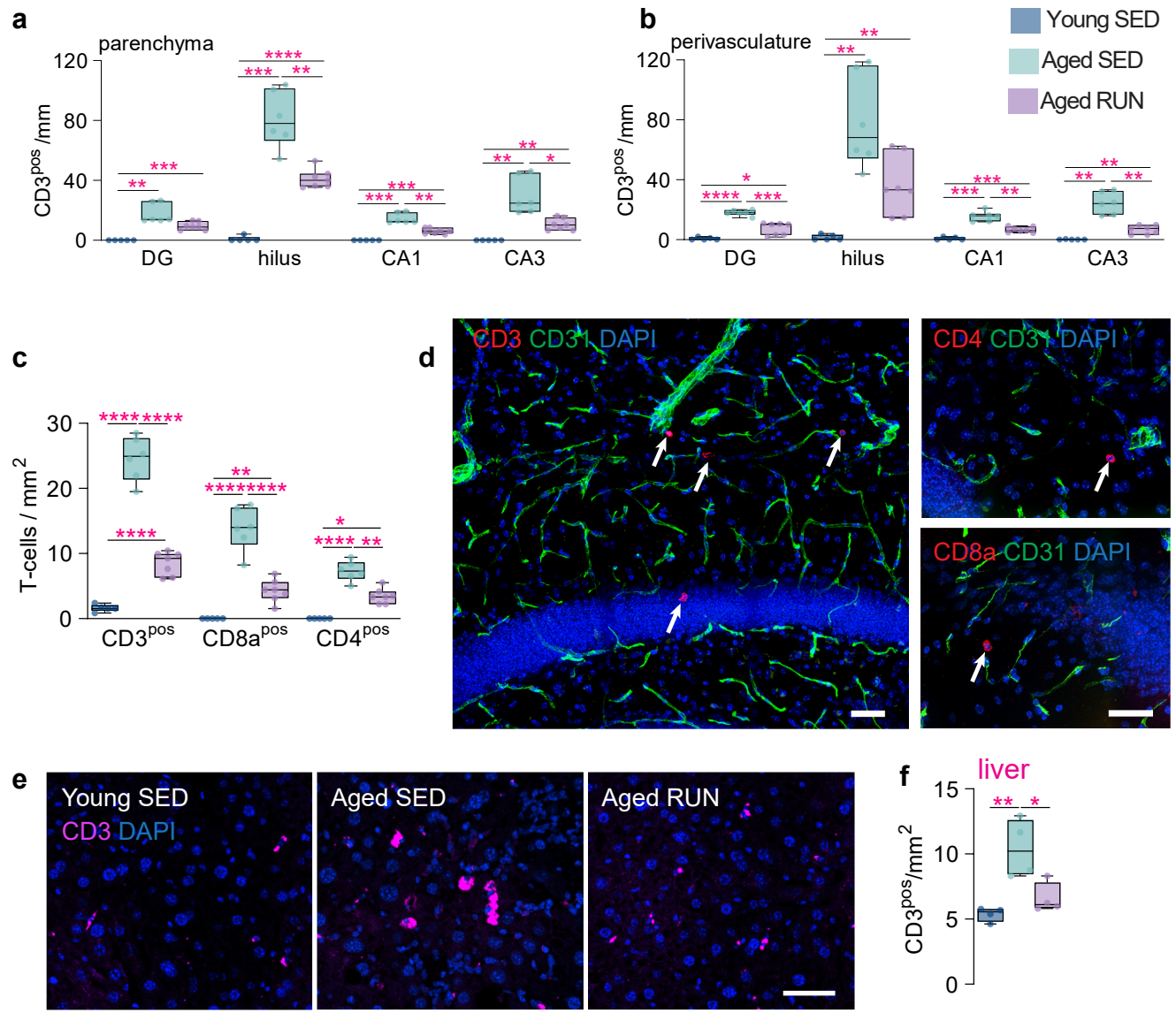

Supplement: Supplementary file 3 — Figure S3. [file ACEL-23-e14172-s007.pdf]

Supplementary Figure 4 (Information for figure 2)

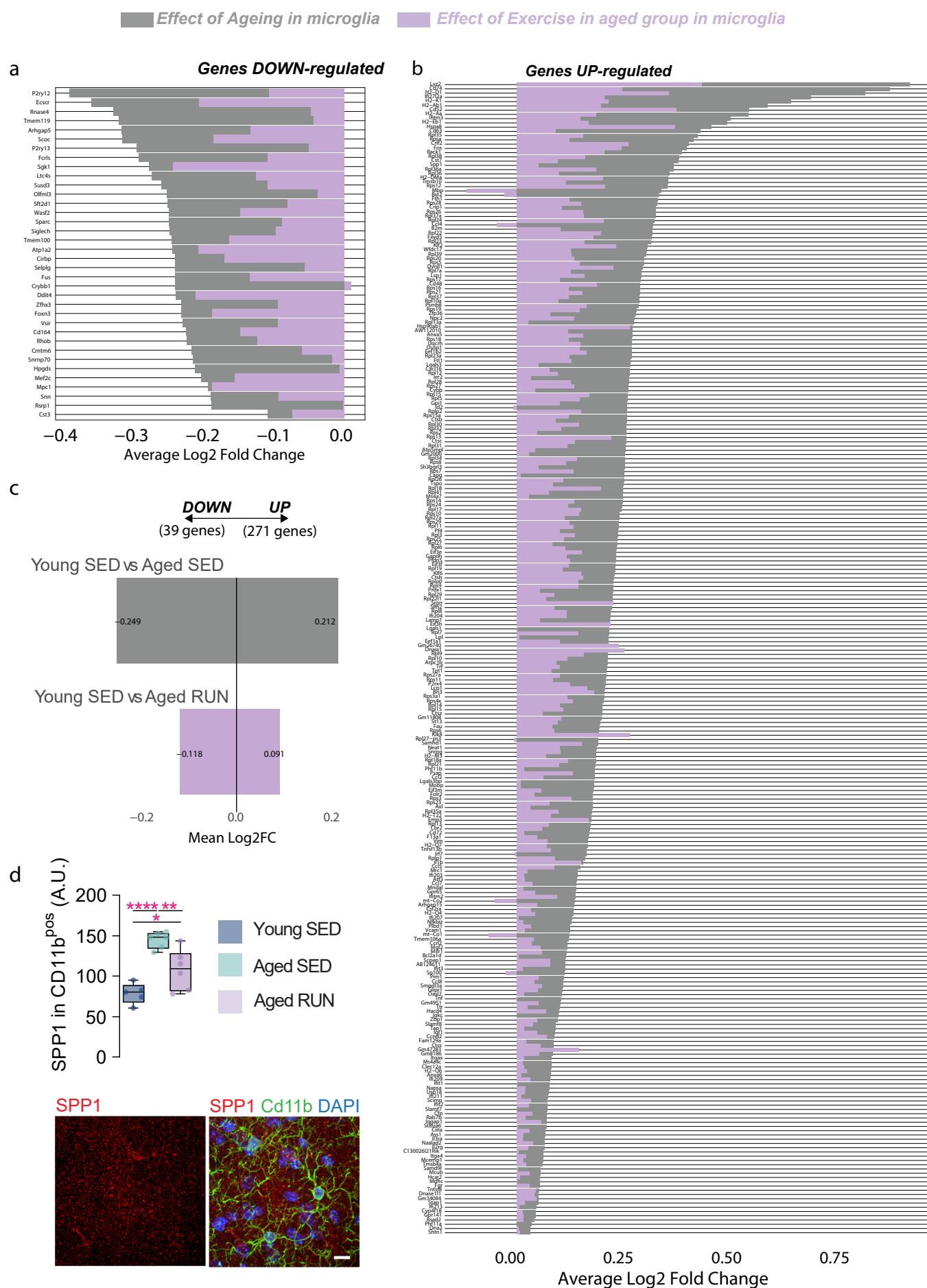

Supplement: Supplementary file 4 — Figure S4. [file ACEL-23-e14172-s008.pdf]

Supplementary Figure 5 (Information for figure 2)

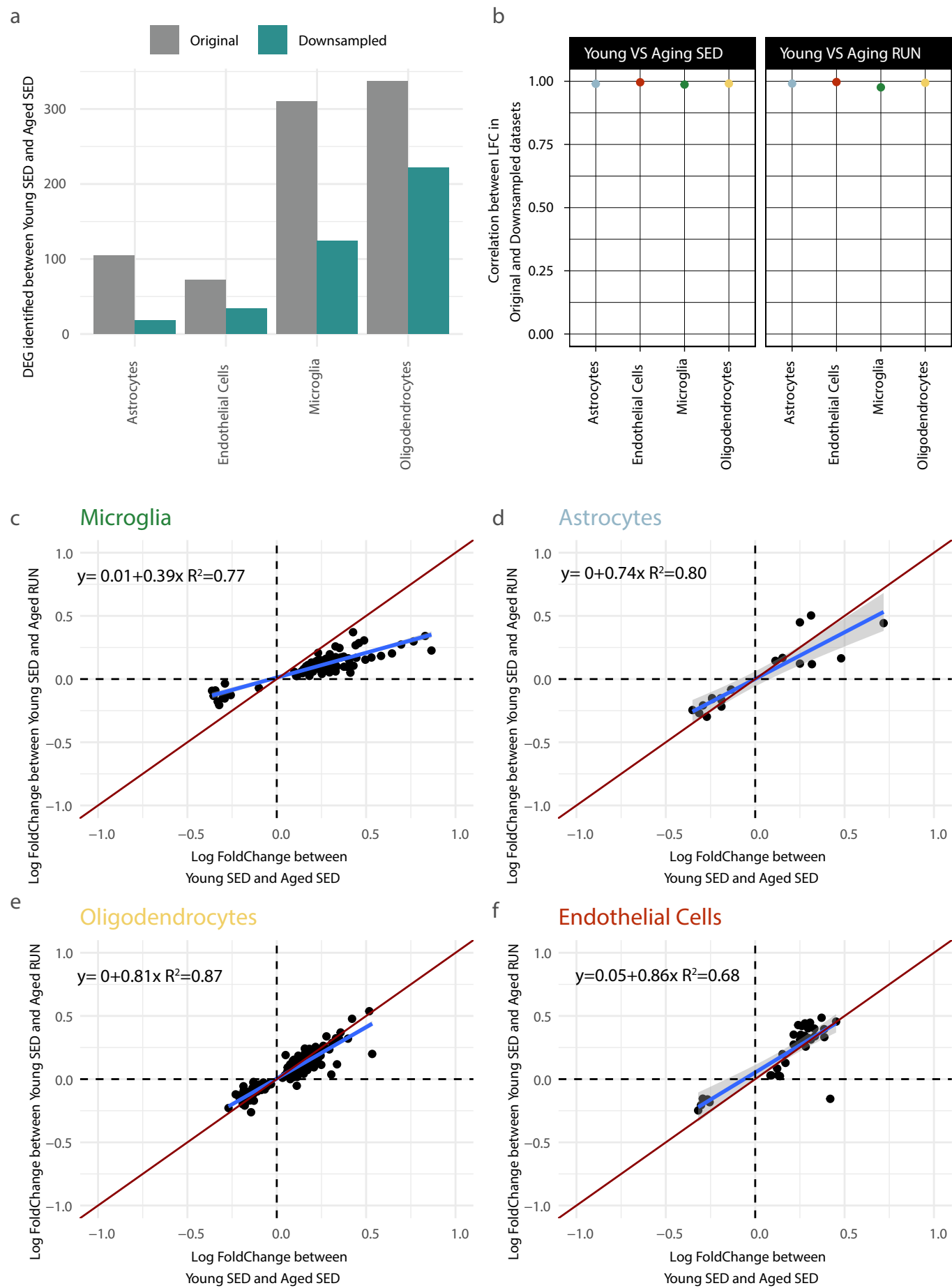

Supplement: Supplementary file 5 — Figure S5. [file ACEL-23-e14172-s002.pdf]

Supplementary Figure 6 (Information for figure 2)

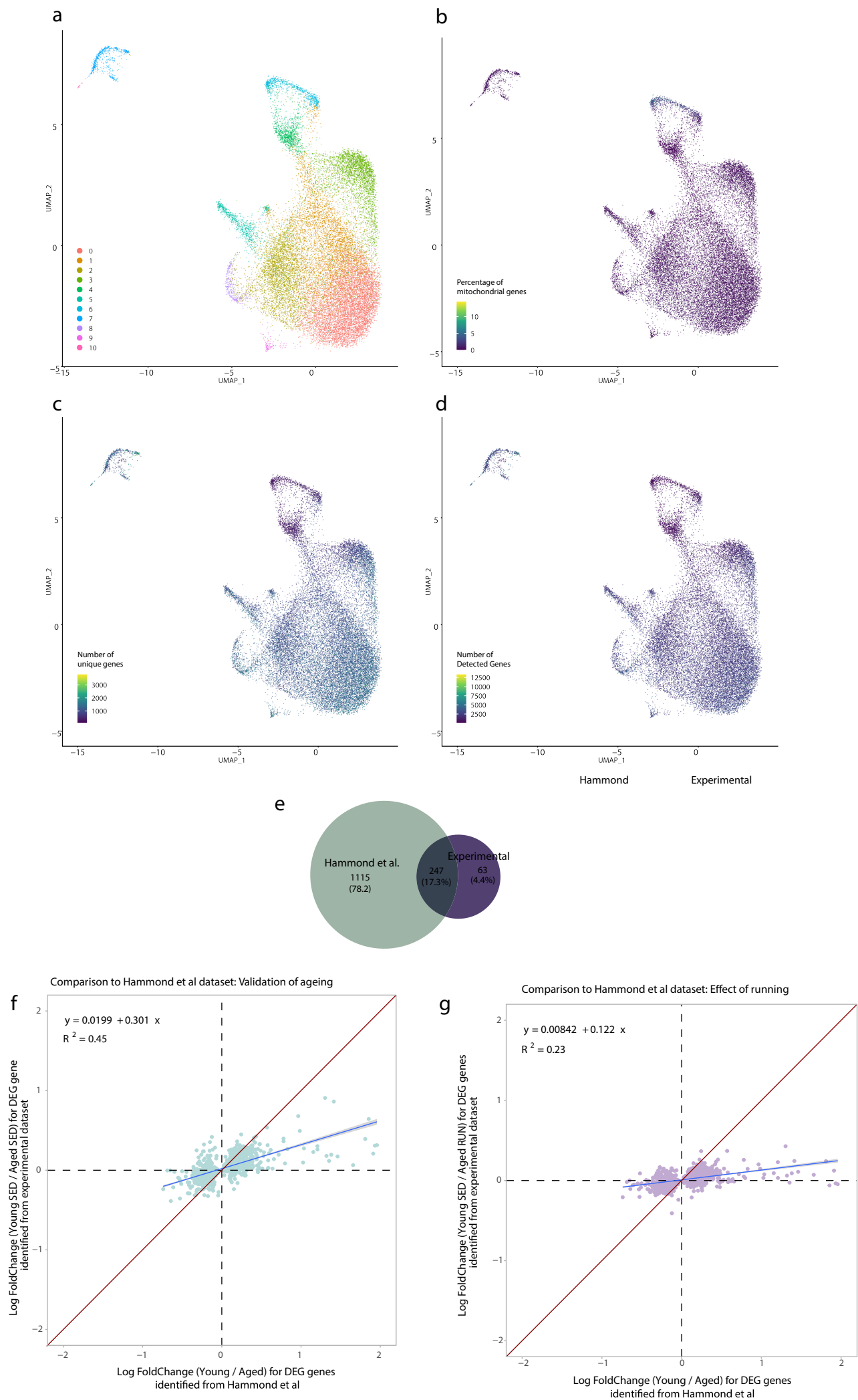

Supplement: Supplementary file 6 — Figure S6. [file ACEL-23-e14172-s003.pdf]

Supplementary Figure 7 (Information for figure 3)

a

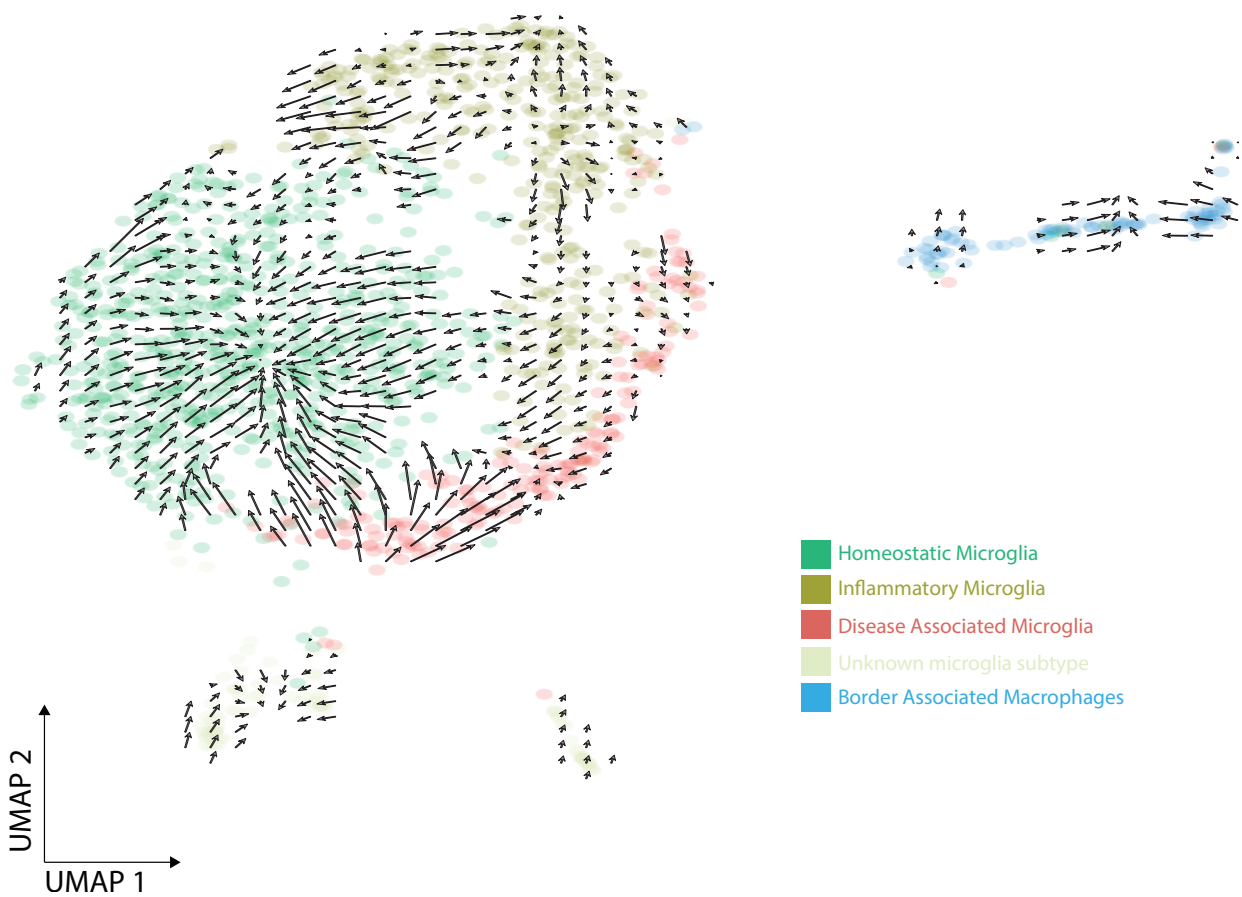

Supplement: Supplementary file 7 — Figure S7. [file ACEL-23-e14172-s009.pdf]

Supplementary figure 8 (Information for figure 4)

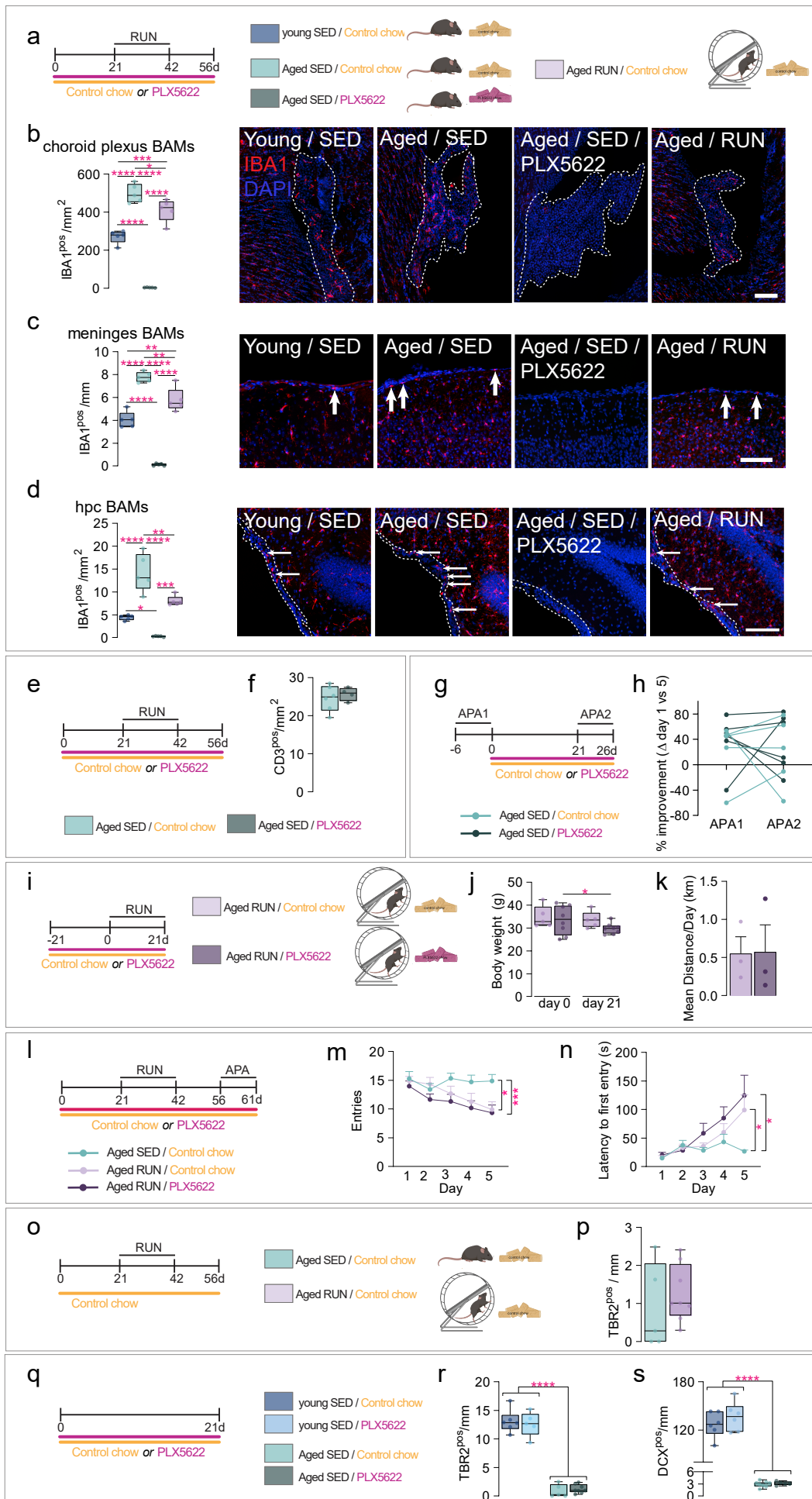

Supplement: Supplementary file 8 — Figure S8. [file ACEL-23-e14172-s006.pdf]
