# Supplementary material for: Exercise rejuvenates microglia and reverses T cell accumulation in the aged female mouse brain
Source: Aging Cell. 2024 May 15;23(7):e14172. doi: 10.1111/acel.14172 (PMC11258432; doi:10.1111/acel.14172)
Supplement: Supplementary file 9 — Data S1. [file ACEL-23-e14172-s001.zip › Chauquet Willis_Supplementary Figure Legends_final.docx]

**Figure S1**: Quality control metrics and cluster evidence for single-cell RNAseq dataset of young adult and aged mouse hippocampus. **Related to Figure 1b.**

**a**) UMAP plot of identified cell clusters in the dataset.

**b**) Violin plot showing the percentage of mitochondrial genes per cell. Cells with a library containing >15% mitochondrial genes were considered abnormal and removed from any further analysis.

**c**) Violin plot showing the number of unique genes detected per cell.

**d**) Violin plot showing the total number of genes detected per cell.

**Figure S2**: Transcriptional signature of T cells in the aged mouse hippocampus. **Related to Figure 1e**.

**a**) UMAP plots showing *Cd4^+^* (left) and *Cd8^+^* (right) T cell populations across all conditions (top), in aged sedentary mice (Aged SED; middle), and aged mice with interventional exercise (Aged RUN; bottom).

**b**) UMAP plots (all T cells) showing expression of *Cd3* subunits, *Cd4*, and additional characteristic markers *Gzmb* and *Prf1* for *Cd8a*-expressing cytotoxic T cells. Both subsets bear the transcriptional signature of tissue-resident memory T cells based on their expression of *Cd69*, *Itgae*, *Cd44*, *Ccr7*, *Sell*, *Cxcr3*, *Klf2*, *Klrg1*, *S1pr1* and *Eomes*. Expression of genes involved in T cell activation (*Cd27* and *Cd28*) and modulation of T cell receptor signalling (*Cd5*) are also shown.

**Figure S3**: T-cells in the aged mouse hippocampus and liver are reduced by exercise *in vivo.* **Related to Figure 1e.**

**a**) Quantification of intraparenchymal CD3^pos^ T-cells in specified hippocampal sub-regions of young and aged sedentary (SED) mice, as well as those with access to a running wheel (Aged RUN mice).

**b**) Quantification of perivascular CD3^pos^ T-cells.

**c**) Quantification of CD3^pos^, CD8a^pos^ and CD4^pos^ T-cells in the hippocampal parenchyma of Young SED, Aged SED, and Aged RUN mice. Counts are from the same mice as shown in Figure 1e.

**d**) Representative confocal images of CD3^pos^, CD4^pos^ and CD8^pos^ T-cells in the hippocampus of Aged sedentary mice; vascular structures were stained for CD31.

**e**) Representative confocal images of CD3^pos^ T-cells in the liver of Young SED, Aged SED, and Aged RUN mice.

**f**) Quantification of CD3^pos^ T-cells in the liver of Young SED, Aged SED, and Aged RUN mice.

Data are represented as box and whisker plots, and dot points show individual mice. Statistics: one-way ANOVA with Bonferroni post-hoc comparisions (a-c, f). Dots represent individual mice. *p<0.05, **p<0.01, ***p<0.001, ****p<0.0001.

**Figure S4:** Exercise reverses the effect of aging in microglia. **Related to Figure 2.**

**a,b**) Bar plots showing the log2FC in gene expression for Aged SED (light grey) and Aged RUN (purple) compared to Young SED microglia. Genes represented are differentially expressed between Aged SED and Young SED, and either significantly down- (a) or up-regulated (b) within this comparison.

**c**) Bar chart representing the average Log_2_FC of up- and down- regulated genes in Aged SED and Aged RUN compared to Young SED. A diminished mean score can be observed for both up- and down- regulated genes in the Young SED vs Aged RUN comparison.

**d**) Validation and quantification (top) of SPP1 protein in CD11b^pos^ cells, showing an increase in SPP1 staining with ageing that is attenuated by exercise. Representative confocal image of immunofluorescent staining showing SPP1 and CD11b^pos^ microglia in the aged hippocampus. Scale bar = 50 µm.

**Figure S5:** Downsampled analysis confirming the effects of ageing and exercise on the microglial transcriptome. **Related to Figure 2.**

1. Bar plot showing the number of differentially expressed genes (DEGs) identified between Young SED and Aged SED mice, using either the total (grey) or downsampled (green) number of cells per cell type to match these between conditions.
2. Correlation between log fold changes (LFC) before and after down-sampling for Young SED and Aged SED (left) and Young SED and Aged RUN (right).

**c-f)** Log fold change comparisons of gene expression in microglia (c), astrocytes (d), oligodendrocytes (e), endothelial cells (f) after down-sampling. Each dot represents the log fold change of one of the DEGs identified between Young SED and Aged SED mice when the number of cells is matched. The red line represents the equation x=y. The blue line corresponds to the best fit of the linear regression following the equation: log fold change (Young SED/Aged RUN) ~ log fold change (Young SED/Aged SED). Shaded area shows the 95% confidence interval of the fitted values.

**Figure S6**: Computational validation of the effect of exercise on aging using a reference scRNA-seq dataset of microglia isolated from young (post-natal day 100) and aged (post-natal day 540) mice from Hammond et al., 2019.^25^ **Related to Figure 2.**

**a**) UMAP plot showing the identified cell clusters from Hammond et al.^25^

**b**) UMAP plot showing the percentage of mitochondrial genes per cell; a threshold of 15% was used due to the brain origin of the sample. Cells with a library containing >15% mitochondrial genes were considered damaged and removed.

**c**) UMAP plot showing the detected number of unique genes per cell across clusters.

**d**) UMAP plot showing the total number of genes detected per cell.

**e**) Venn diagram showing overlap between markers of ageing (DEGs) identified in the Hammond et al. dataset (green) and those identified in the current study (purple). Markers of ageing identified in our dataset were also significantly enriched within the markers of ageing identified in the Hammond et al. dataset (Fisher’s exact test, p = 2.00x10^-199^).

**f**) Log fold change calculated for DEGs between young and aged mice in the Hammond et al. dataset, and Young SED and Aged SED in the current study; note that ageing-related DEGs behaved similarly in both datasets. Each dot represents one of the 1362 differentially expressed genes (young vs aged) identified in the Hammond et al. dataset. The dark red line represents the equation y=x. The blue line corresponds to the best fit of the linear regression, following the equation: log foldchange (Young SED/Aged SED) ~ log fold change (young/aged); the shaded area shows the 95% confidence interval of the fitted values. Coefficients, standard error and R^2^ for the linear regression are the following: 0.301±0.009, R^2^=0.45.

**g**) Log fold change calculated for DEGs between young and aged mice in the Hammond et al dataset, and Young SED and Aged RUN in the current study, showing an effect of exercise on ageing-related DEGs. Each dot represents one of the 754 markers of ageing identified in the Hammond dataset. The red line represents the equation x=y. The blue line corresponds to the best fit of the linear regression following the equation: log fold change (Young SED / Aged RUN) ~ log fold change (young/aged); the shaded area shows again the 95% confidence interval of the fitted values. Coefficients, standard error and R^2^ for the linear regression are the following: 0.122±0.006, R^2^=0.23.

**Figure S7:** RNA velocity analysis of aged microglial subtypes in response to exercise. **Related to Figure 3.**

**a)** RNA velocity analysis of microglial subtypes and/or states in response to exercise, showing a preferential transition of inflammatory and disease-associated microglia towards the homeostatic state (note the direction of longer arrows).

**Figure S8**: *In vivo* validation of BAMs and T-cells, and additional measures of exercise, cognition and hippocampal neurogenesis. **Related to Figure 4.**

**a)** Overview of experimental timeline. Aged (18-month-old) mice were placed on PLX5622-containing chow (to deplete microglia), or control chow, and allowed access to a running wheel for 21 days, followed by 2 weeks rest.

**b-d**) Quantification (left) and confocal images of border associated macrophages (BAMs; right) in the choroid plexus (b), meninges (c), and perivascular spaces (d) of the hippocampus of experimental mice. Scale bar = 100 µm.

**e)** Experimental timeline for Aged (18-month-old) mice placed on PLX5622-containing chow (to deplete microglia), or control chow.

**f)** Quantification of CD3-positive T-cells in the parenchyma of the hippocampus of Aged SED mice treated with either control chow or PLX5622 (microglial depletion).

**g)** Overview of experimental timeline. Mice underwent active place avoidance (APA) testing over a course of 5 days (APA1). Aged mice were then split into PLX5622-containing chow (to deplete microglia) or control chow groups, and then re-tested for acquisition of a new APA task (APA2) 21 days later.

**h)** Percentage improvement in APA performance for individual mice, assessed by the change in entries on testing day 5 versus testing day 1; data points indicate individual mice and the connecting lines shows their performance in APA1 and APA2.

**i**) Overview of experimental timeline for aged (18-month-old) mice, placed on PLX5622-containing chow (to deplete microglia), or control chow, and allowed access to a running wheel for 21 days.

**j**) Body weight of aged mice with and without microglia depletion, at the beginning (day 0) and end (day 21) of the exercise paradigm.

**k**) Mean distance ran per day for aged mice treated with either control or PLX5622 chow.

**l**) Experimental timeline for aged (18-month-old) mice, placed on PLX5622-containing chow (to deplete microglia), or control chow, and allowed access to a running wheel for 21 days. Mice were assessed for APA task acquisition two weeks after the conclusion of exercise.

**m)** Total number of entries into the shock zone during APA testing (20-min trials/day; downsampled to the first 10 minutes only).

**n)** Latency to first enter the shock zone over five testing days in the APA task.

**o)** Experimental timeline for Aged (18-month-old) mice allowed access to a running wheel for 21 days.

**p)** Quantification of TBR2^pos^ intermediate neuronal progenitors in Aged SED / control chow and Aged RUN / control chow mice.

**q)** Experimental timeline for young (3-month-old) and aged (18-month-old) mice fed control or PLX5622-containing chow.

**r)** Quantification of TBR2^pos^ intermediate neuronal progenitors in young (3-month-old) and aged (18-month-old) mice, with microglia depletion.

**s)** Quantification of DCX^pos^ immature neurons in young (3-month-old) and aged (18-month-old) mice, with microglia depletion.

Data are represented as mean ± SEM. Statistics: one-way ANOVA (b-d), repeated two-way ANOVA (j,m,n), unpaired (f,k,p) or paired (h) Student’s t-test, or two-way ANOVA (r,s) followed by Bonferroni post-hoc comparison with Geisser-Greenhouse correction. *p<0.05, **p<0.01, ***p<0.001, ****p<0.0001.
