# Supplementary material for: Exercise rejuvenates microglia and reverses T cell accumulation in the aged female mouse brain
Source: Aging Cell. 2024 May 15;23(7):e14172. doi: 10.1111/acel.14172 (PMC11258432; doi:10.1111/acel.14172)
Supplement: Supplementary file 9 — Data S1. [file ACEL-23-e14172-s001.zip › Figure_SupplementaryNote_submitted.docx]

**Supplementary Note – Data S1**

***Differential effects of exercise on the transcriptome of ageing brain cell populations other than microglia***

*Astrocytes.* For astrocytes, we observed a larger number of transcriptional differences in the Young SED vs Aged SED condition (105 DEGs; **Table S7**) than in Young SED vs Aged RUN (63 DEGs; **Table S8**), which represented a 40% reduction in the number of DEGs between young and aged mice when the latter are exposed to exercise (**Figure 2a**). This suggests that exercise may reduce, at least in part, some of the effects of ageing in astrocytes. However, the magnitude of reversal of age-related gene expression changes in the presence of exercise appeared relatively subtle, with a regression coefficient of 0.739x±0.042 (R^2^=0.75) (see below in **Data S1a**). Genes involved in oxidative phosphorylation and electron transport chain were significantly enriched in ageing astrocytes, a phenotype that was even stronger with interventional exercise (Oxidative phosphorylation: Young SED vs Aged SED: FDR corrected p=3.32x10^-05^; Young SED vs Aged RUN: FDR corrected p=6.91x10^-13^; Electron transport chain: Young SED vs Aged SED: FDR corrected p= 1.48x10^-06^; Young SED vs Aged RUN: FDR corrected p=2.75x10^-25^). Genes in the anti-inflammatory PPAR signalling pathway (FDR corrected p =2.196x10^-02^), and for cytoplasmic ribosomal proteins (FDR corrected p = 2.89x10^-02^) were only enriched in the presence of exercise (see below **Data S1d**).

*Endothelial cells*. For endothelial cells, a total of 72 DEGs were identified between Young SED and Aged SED mice (**Table S9**), and 109 DEGs were identified between the Young SED and Aged RUN (**Table S10**; **Figure 2a**). A large proportion of these age-related gene expression changes (43 out of 72 DEGs; 59%) remained significant in the exercise condition. When comparing the fold changes for these DEGs between Young SED vs Aged SED with those observed in the Young SED vs Aged RUN, we again observed only subtle effects of exercise on the ageing-phenotype, with a regression coefficient close to 1 (0.935x±0.062, R^2^=0.77; see below **Data S1b**). The MAPK signaling pathway was significantly regulated in both aging conditions (Young SED vs Aged SED: FDR corrected p= 1.31x10^-02^; Young SED vs Aged RUN: FDR corrected p= 9.23x10^-3^; see below **Data S1d**). Endothelial cells exhibit a more active metabolic profile overall in the presence of exercise, as demonstrated by enrichment of genes involved in glycolysis and gluconeogenesis (FDR corrected p=1.61x10^-04^; see below **Data S1d**), which play an important role in eliciting cellular responses to changing environmental conditions.

*Oligodendrocytes.* Comparing oligodendrocytes between Young SED and Aged SED identified 337 DEGs (**Table S11**). A total of 278 DEGs were identified between Young SED and Aged RUN condition (**Table S12**), with 57% (195 out of 337 genes) of age-related DEGs remaining significant in the presence of exercise (**Figure 2a**). Comparing the expression fold changes for Young SED vs Aged SED with those observed in Young SED vs Aged RUN, we again only observed relatively subtle effects of exercise on these cells, with a regression coefficient close to 1 (0.813x±0.017, R^2^=0.87) (see below **Data S1c** ). Pathway enrichment (see below **Data S1d**) identified mRNA processing as the only pathway that was significantly enriched in the Young SED vs Aged SED gene set (FDR corrected p=1.21x10^-04^); this pathway was not significant when exercise was introduced to the aging condition. Four different pathways were significant with the introduction of exercise. Those pathways were related to energy generation (Electron transport chain: FDR corrected p=2.90x10^-03^, Exercise-induced circadian regulation: FDR corrected p=3.34x10^-02^), innate immunity (Complement activation classical pathway: FDR corrected p=8.81x10^-04^), and oxidative stress (Oxidative damage response: FDR corrected p=1.51x10^-02^).

In summary, although all cell types exhibited differential gene expression with ageing, DEGs from endothelial cells and oligodendrocytes were associated with only a small number of pathways. In astrocytes, ageing was associated with an enrichment of DEGs involved in oxidative phosphorylation and the electron transport chain, suggesting age-related alterations in metabolic activity and/or mitochondrial dynamics. This is consistent with prior studies reporting a lower expression of genes involved in mitochondrial function and energy production (*Ucp2, Cox8b and Atp5g1*) in aged hippocampal astrocytes^1^. Interestingly, exercise resulted in additional astrocytic DEGs in aged mice, with enrichments in the peroxisome proliferator-activated receptor (PPAR) signalling and selenium micronutrient pathways. PPAR agonists can mitigate CNS disease by modulating inflammatory signalling in immune cells^2^ and astrocytes^3^, while, intriguingly, the antioxidant selenium has recently been identified as a systemic factor involved in mediating the exercise-induced increase in hippocampal neurogenesis^4^. As our results indicate that the astrocytic selenium pathway is influenced by exercise in the aged brain, and given that astrocytes are major regulators of adult neurogenesis^5^, it would be interesting to determine whether the exercise-induced influx of selenium into the brain acts via astrocytes to enhance hippocampal neurogenesis.

**References**

1. Clarke, L. E. *et al.* Normal aging induces A1-like astrocyte reactivity. *Proc. Natl. Acad. Sci. U. S. A.* **115**, E1896–E1905 (2018).

2. Daynes, R. A. & Jones, D. C. Emerging roles of PPARs in inflammation and immunity. *Nat. Rev. Immunol.* **2**, 748–759 (2002).

3. Iglesias, J., Morales, L. & Barreto, G. E. Metabolic and Inflammatory Adaptation of Reactive Astrocytes: Role of PPARs. *Mol. Neurobiol.* **54**, 2518–2538 (2017).

4. Leiter, O. *et al.* Selenium mediates exercise-induced adult neurogenesis and reverses learning deficits induced by hippocampal injury and aging. *Cell Metab.* **34**, 408-423.e8 (2022).

5. Cassé, F., Richetin, K. & Toni, N. Astrocytes’ Contribution to Adult Neurogenesis in Physiology and Alzheimer’s Disease. *Front. Cell. Neurosci.* **12**, 432 (2018).


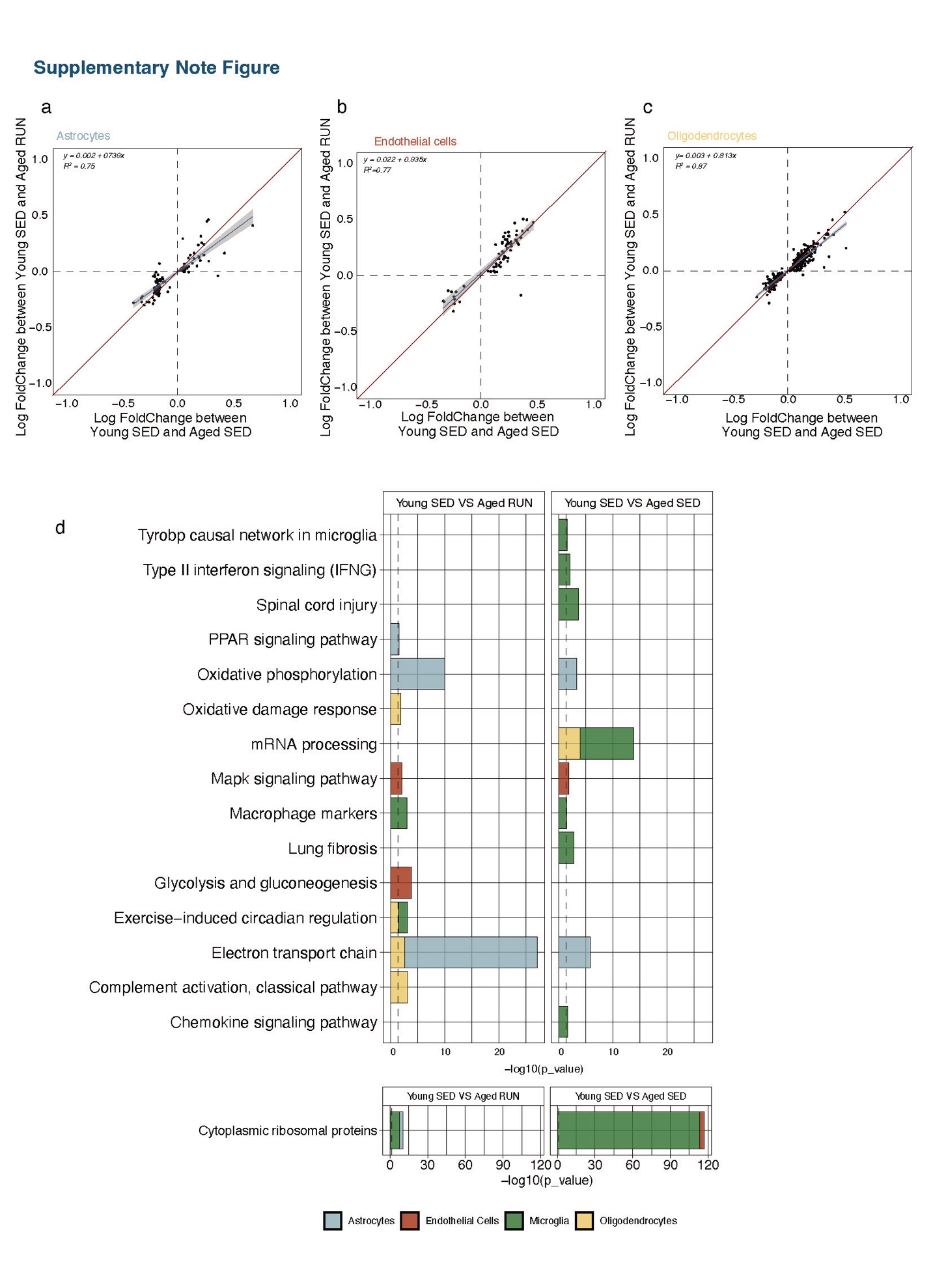
**Supplementary Note figure – Data S1**

**Supplementary Note figure – Data S1:**

**a-c)** Log foldchange comparisons of gene expression in astrocytes (**c**), endothelial cells (**d**), and oligodendrocytes (**e**). Each dot represents the log fold change of one of the differentially expressed genes identified between Young SED and Aged SED mice. The red line represents the equation x=y. The blue line corresponds to the best fit of the linear regression following the equation: log foldchange (Young SED/Aged RUN) ~ log foldchange (Young SED/Aged SED). Shaded area shows the 95% confidence interval of the fitted values. Coefficients, standard error and R^2^ for the linear regression are the following: 0.739x±0.042, R^2^=0.75 (astrocytes); 0.935x±0.062, R^2^=0.77 (endothelial cells); and 0.813x±0.017, R2=0.87 (oligodendrocytes).

**d)** Enriched pathways in astrocytes, endothelial cells, oligodendrocytes, and microglia between experimental conditions. Dashed lines show significance threshold of -log_10_(0.05).
